# Microbial Signatures Associated with Oropharyngeal and Oral Squamous Cell Carcinomas

**DOI:** 10.1038/s41598-017-03466-6

**Published:** 2017-06-22

**Authors:** Sagarika Banerjee, Tian Tian, Zhi Wei, Kristen N. Peck, Natalie Shih, Ara A. Chalian, Bert W. O’Malley, Gregory S. Weinstein, Michael D. Feldman, James Alwine, Erle S. Robertson

**Affiliations:** 1 Department of Otorhinolaryngology-Head and neck surgery, University of Pennsylvania, Philadelphia, Pennsylvania 19104 United States of America; 2 Department of Computer Science, New Jersey Institute of Technology, New Jersey, 07102 United States of America; 3 Department of Pathology and Laboratory Medicine, University of Pennsylvania, 19104 Philadelphia, Pennsylvania United States of America; 4 Department of Cancer Biology, University of Pennsylvania, Philadelphia, Pennsylvania 19104 United States of America

## Abstract

The microbiome is fundamentally one of the most unique organs in the human body. Dysbiosis can result in critical inflammatory responses and result in pathogenesis contributing to neoplastic events. We used a pan-pathogen array technology (PathoChip) coupled with next-generation sequencing to establish microbial signatures unique to human oral and oropharyngeal squamous cell carcinomas (OCSCC/OPSCC). Signatures for DNA and RNA viruses including oncogenic viruses, gram positive and negative bacteria, fungi and parasites were detected. Cluster and topological analyses identified 2 distinct groups of microbial signatures related to OCSCCs/OPSCCs. Results were validated by probe capture next generation sequencing; the data from which also provided a comprehensive map of integration sites and chromosomal hotspots for micro-organism genomic insertions. Identification of these microbial signatures and their integration sites may provide biomarkers for OCSCC/OPSCC diagnosis and prognosis as well as novel avenues for study of their potential role in OCSCCs/OPSCCs.

## Introduction

Cancer remains the second most common cause of death in the US preceded by heart disease, accounting for nearly 1 of every 4 deaths^1^. Oral cancer (includes oral cavity and oropharyngeal cancers) is one of the most common cancers worldwide, and incidence rates are higher in men compared to women^1–3^. The predicted new oral cancer cases in 2016 will be 48,250 in the US, with predicted new cases annually exceeding 450,000, worldwide^1, 2^. Oral cancer is newly diagnosed in about 115 new individuals each day in the US alone, and 1 person dies from the disease every hour. Oral squamous cell carcinoma (OSCC) is the most common oral cancer, comprising about 90% of all the oral cancers^4^. In the US, 3% of cancers in men and 2% in women are OSCC, most of which occur after age 50^5^. The majority of cases are diagnosed at the late stage of cancer, and this accounts for the very high death rate of about 50% at five years from diagnosis^2^. However, if diagnosed at early stages of development, the survival rate for oral cancer is relatively high at 80–90%^2^. A 70–80% risk factor for oral cancer has been linked to tobacco and alcohol usage^6^ and more recently about 20% to 25% to HPV16 infection^2^. Less than 7% of oral cancers are not linked to a specific cause and can be attributed to genetic susceptibility^2^ or to infections or dysregulation of the oral microbiome^7–9^.

The 5 year survival post diagnosis of OSCC is directly related to the stage at diagnosis. Therefore, early detection efforts have the potential to increase the survival rate. Notably, during the early stage, oral cancer lesions can go unnoticed, as it is asymptomatic and painless^2^. Thus discovering biomarkers for oral cancer will be useful for early diagnosis and increased survival rate. However, as of today there are no efficient biomarkers for oral cancer^10^. Studies focused on associating bacterial flora with oral cancer have suggested that some salivary bacteria may be indicators of disease, which is potentially useful in patient diagnosis, monitoring, and overall health evaluation^7, 11–13^. However, 35% to 50% of the oral microbiome is uncultivable, leaving no way to determine the association with oral health or disease^11, 14–16^. Most independent laboratory techniques, including next generation sequencing (NGS), bacterial microarrays, DNA hybridization, PCR, and quantitative PCR, are currently used to determine the association of bacteria with oral health and disease, but not as a diagnostic tool^11, 17^. A significant change in the oral microbial environment may provide clues for identification of oral cancer specific microbial biomarkers. For the microbes to be considered disease-specific biomarkers (microbial biomarkers), they must be associated directly with the condition in question, but not necessarily the cause^11, 18^.

We have used a pan-pathogen array technology called PathoChip coupled with a capture next-generation sequencing strategy, to identify the microbial signatures associated with oropharyngeal (OPSCC) or oral cavity (OCSCC) squamous cell carcinomas. The array is comprised of oligonucleotide probes that can detect all sequenced viruses, as well as human pathogenic bacteria, fungi and parasites. Additionally, it contains, family-specific conserved probes which provide a means for detecting previously uncharacterized members of a family. We have previously used PathoChip to define viral and other microbiome signatures in triple negative breast cancer^19^. In this present studies we analyzed the specific viral, bacterial, fungal and parasitic microbial signatures specifically associated with tissues obtained from OCSCCs which were predominantly oropharyngeal with some number of buccal and tongue based cancers. We have collectively referred to them as OCSCC/OPSCC, and henceforth in the manuscript will refer them as OCSCC. The microbiome signatures found in OCSCC tissue were quite different from signatures found in adjacent clinically normal controls or oral tissue from otherwise healthy controls. Interestingly, a predominant HPV16 genetic signature was found associated specifically with the OCSCC samples. These studies have now identified potential microbial signatures unique for oral cavity squamous cell carcinoma using the PathoChip platform.

The PathoChip results were validated using specific positive probes to capture pathogenic targets from the cancer samples. These were subjected to next generation sequencing (NGS) to determine the identification of the pathogen detected by the array. To enhance our understanding of the role of these pathogens in OCSCC, we used the NGS data to determine if there were sites of viral or microbial DNA integration into the host genome. Integration hotspots for HPV16 were identified along with other identified integration sites for a number of viruses, including the JC polyomavirus, as well as other pathogenic and tumorigenic bacteria, fungi and parasites in these OCSCC samples. Our data strongly suggest greater molecular intimacy between the host genome and genomes of associated microbial agents in the tumor microenvironment.

## Results

### Microbial signatures detected in OCSCCs

Using the PathoChip technology we screened 100 FFPE pathologically defined OCSCC patient samples as well as 20 cancer adjacent normal controls (matched) and 20 oral tissue (uvula) from healthy individuals (non-matched controls) for distinct viral and microbial signatures associated with the tumor tissue. Samples analyzed in this study were carcinomas taken from tongue, base of tongue, tonsil, floor of mouth, cheek and predominantly oropharynx which we refer to as OCSCC in this study (Table S1). To identify the microbial signatures associated with OCSCC, both DNA and RNA were extracted from the samples, subjected to whole genome and transcriptome amplification (referred here as WGTA), labelled and hybridized to the probes on the PathoChip.

#### A. Viral signatures associated with OCSCC

**Figure 1:**
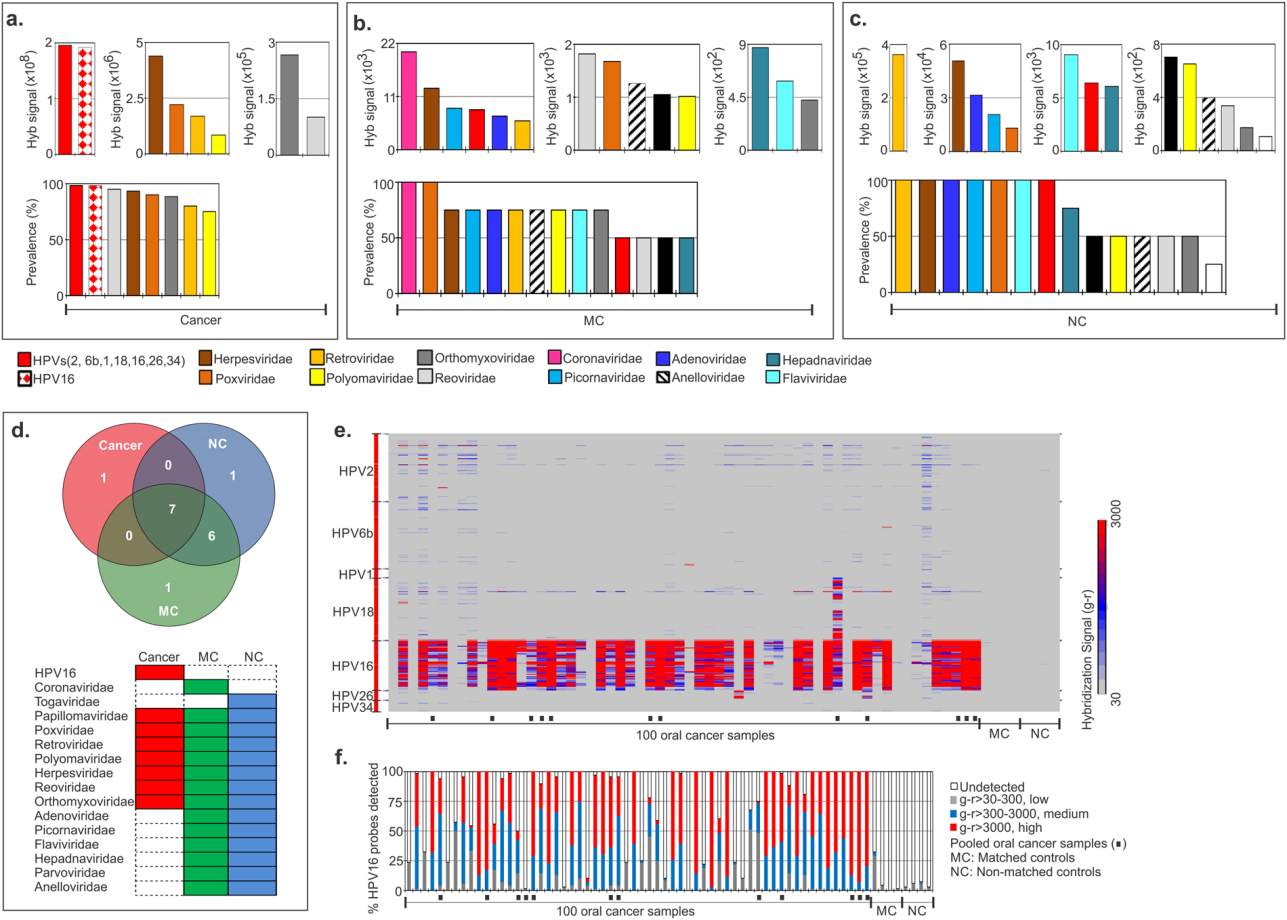
Viral signatures detected in oral cancer and control samples. (**a**) The viral signatures that are detected with hybridization signal (g–r > 30) by PathoChip screen of 100 oral cancer samples are shown and ranked according to decreasing hybridization signal (weighted score sum of all the probes per accession) and prevalence. (**b** and **c**) Figure **b** and **c** shows the hybridization signals and prevalence for the viral signatures detected in matched (MC) and non-matched (NC) controls respectively, ranked in descending order. (**d**) Figure **d** shows the association of different molecular signatures of viral families with cancer and controls, represented as a venn diagram, and as colored bars. (**e**) Figure **e** shows the heat map of hybridization signals detected by PathoChip screen of the HPV probes (Y-axis) with the oral cancer and control samples (x-axis). The hybridization signals of the cancer samples to each of these probes were compared to MCs and NCs. Samples were screened individually or in pools (marked with a ▪). (**f**) Figure **f** shows percentage of HPV16 probes detected with low (g-r > 30–300), medium (g–r > 300–3000) and high (g–r > 3000) hybridization signal in 100 oral cancer samples screened individually and in pools (▪) and 20 each of MCs and NCs screened in pools of 5.

We identified RNA and DNA viruses associated with the cancer and control samples (Fig. 1 and Table S2). Viral sequences belonging to Papillomaviridae showed the highest hybridization signal in the OCSCC samples screened, followed by that of Herpesviridae, Poxviridae, Retroviridae and Polyomaviridae (Fig. 1a). Viral signatures belonging to all of these families were seen to be >75% prevalent among the 100 OCSCC samples screened. Interestingly, Papillomaviridae was detected in 98% of the cases (Fig. 1a). The hybridization signal for all papillomaviruses was much higher in the OCSCC samples compared to the matched and non-matched controls (Fig. 1a,b,c,e and Table 1). Importantly, HPV16 was detected with both high hybridization signal and prevalence (98%) only in the OCSCC samples (Fig. 1a,d,e and f). Figure 1f shows that nearly all of HPV16 specific probes were detected in the majority of OCSCC samples with medium (blue) to high (red) hybridization signal (Fig. 1e and f). In contrast the HPV16 probes were detected with significantly lower (grey) hybridization signals in both matched and non-matched controls (Fig. 1e and f). Signatures of Reoviridae, Herpesviridae, Poxviridae, Orthomyxoviridae, Retroviridae and Polyomaviridae were detected in OCSCC samples with high prevalence and at hybridization signals that were 2–3 logs higher than in controls (Fig. 1a and Table 1). Notably, viral signatures of Coronoviridae, Picornaviridae, Adenoviridae, Anelloviridae, Hepadnaviridae and Flaviviridae were significantly and specifically detected in the controls along with signatures of non-HPV16 papillomaviridae (Fig. 1b and c). These data show that viral signature is significantly changed when compared specifically to the OCSCC tissue.

**Table 1:** Significant detection of the probes of micro-organisms in cancer compared to the matched (MC) and non-matched control (NC) samples.

Types	Phyla	Family/Genera	Hybridization Signal (weighted score sum)			*p*-value	
			Cancer	MC	NC	Cancer vs. MC	Cancer vs. NC
Viruses		HPVs (2, 6b, 1, 18, 16, 26, 34)	195426486	8029	6839	2.31E-10	2.3E-10
		HPV16	191835193	6468	2862	4.02E-10	3.98E-10
		Poxviridae	2206031	3151	11698	9.95E-06	3.59E-05
		Retroviridae	1696995	7616	73887	0.002287	0.291564
		Polyomaviridae	847250	1153	69753	8.59E-05	0.427587
		Herpesviridae	4383363	9881	74503	7.97E-07	9.1E-05
		Reoviridae	101004	44	251	3.26E-09	7.59E-09
		Orthomyxoviridae	266120	235	158	1.4E-05	1.3E-05
Bacteria	Actinobacteria	*Arcanobaterium*	4313000	0	81	8.44E-06	8.48E-06
	Actinobacteria	*Mobiluncus*	2684602	38	229	2.83E-06	2.88E-06
	Actinobacteria	*Actinomyces*	2095762	310	0	1.46E-05	1.41E-05
	Actinobacteria	*Rothia*	1255769	0	0	6.62E-06	6.62E-06
	Actinobacteria	*Propionibacterium*	1192284	0	75	4.33E-06	4.4E-06
	Actinobacteria	*Mycobacterium*	632038	1186	5901	0.000114	0.000808
	Proteobacteria	*Brevundimonas*	662587	0	0	0.007274	0.007274
	Proteobacteria	*Cardiobacterium*	53035	217	0	0.002417	0.001331
	Proteobacteria	*Aeromonas*	412231	4524	478	0.031211	0.00984
	Proteobacteria	*Bordetella*	381307	188	70	0.005386	0.005212
	Proteobacteria	*Comamonas*	372204	2567	435	0.000375	4.83E-05
	Proteobacteria	*Alcaligenes*	347736	1782	166	0.008933	0.005065
	Proteobacteria	*Caulobacter*	327628	37	0	0.002719	0.002682
	Proteobacteria	*Acinetobacter*	297486	168	248	0.012434	0.012716
	Proteobacteria	*Citrobacter*	231427	0	3448	0.030438	0.089252
	Proteobacteria	*Sphingomonas*	206703	0	112	0.02798	0.028969
	Proteobacteria	*Plesiomonas*	117585	31	4521	0.053936	0.311419
	Proteobacteria	*Actinobacillus*	115458	0	0	0.365984	3.95E-06
	Proteobacteria	*Serratia*	111747	0	2046	0.039465	0.125478
	Proteobacteria	*Edwardsiella*	81383	0	0	0.045451	0.045451
	Proteobacteria	*Haemophilus*	45839	0	37	0.015141	0.016169
	Proteobacteria	*Frateuria*	16380	0	36	0.006651	0.006758
	Proteobacteria	*Eshcherichia*	2278234	0	0	4.41E-06	4.41E-06
	Firmicutes	*Peptoniphilus*	846305	0	0	0.000318	0.000318
	Firmicutes	*Aerococcus*	338497	2586	69	0.003597	0.000755
	Firmicutes	*Pediococcus*	321750	288	72	0.000116	0.000101
	Firmicutes	*Peptostreptococcus*	65757	0	0	0.061028	0.060022
	Firmicutes	*Veillonella*	53035	217	0	0.005775	0.003531
	Firmicutes	*Streptococcus*	50613	228	379	0.05041	0.058804
	Bacteroidetes	*Prevotella*	290038	275	338	0.012396	0.012614
	Bacteroidetes	*Sphingobacterium*	352917	187	110	0.000286	0.000275
Fungi		*Fonsecaea*	17241169	27419	62314	3.1E-20	5.16E-18
		*Rhodotorula*	12912140	0	0	2.1E-19	2.1E-19
		*Cladophialophora*	11366446	11539	25248	7.71E-17	1.64E-16
		*Geotrichum*	8982809	0	0	1.42E-11	1.42E-11
		*Malassezia*	7035309	87	4425	5.87E-13	6.64E-10
		*Cladosporium*	4991102	185123	361631	0.052290	0.401344
		*Pleistophora*	2163898	2504	21859	1.11E-16	8.26E-14
		*Pneumocystis*	1210219	0	0	9.75E-08	9.75E-08
		*Absidia*	283315	235	216	5.87E-13	5.67E-13
		*Phialophora*	74254	0	372	5.75E-10	5.01E-09
Parasite		*Hymenolepis*	26463760	0	0	3.15E-29	3.15E-29
		*Centrocestus*	19026989	0	0	3.40901E-29	3.40901E-29
		*Dipylidium*	16438588	2402	65928	1.38087E-19	1.78333E-17
		*Prosthodendrium*	10743239	16756	37938	2.52E-18	9.31E-18
		*Trichinella*	1814992	0	0	5.19E-09	5.19E-09
		*Contracaecum*	306180	0	0	1.19E-16	1.19E-16
		*Toxocara*	260321	0	0	6.87E-15	6.87E-15

Weighted score sum of the hybridization signals of all the probes of an organism was calculated in cancer and controls, and significance (*p*-value < 0.05) was calculated using one sided t-tests.

#### B. Bacterial signatures associated with OCSCC

**Figure 2:**
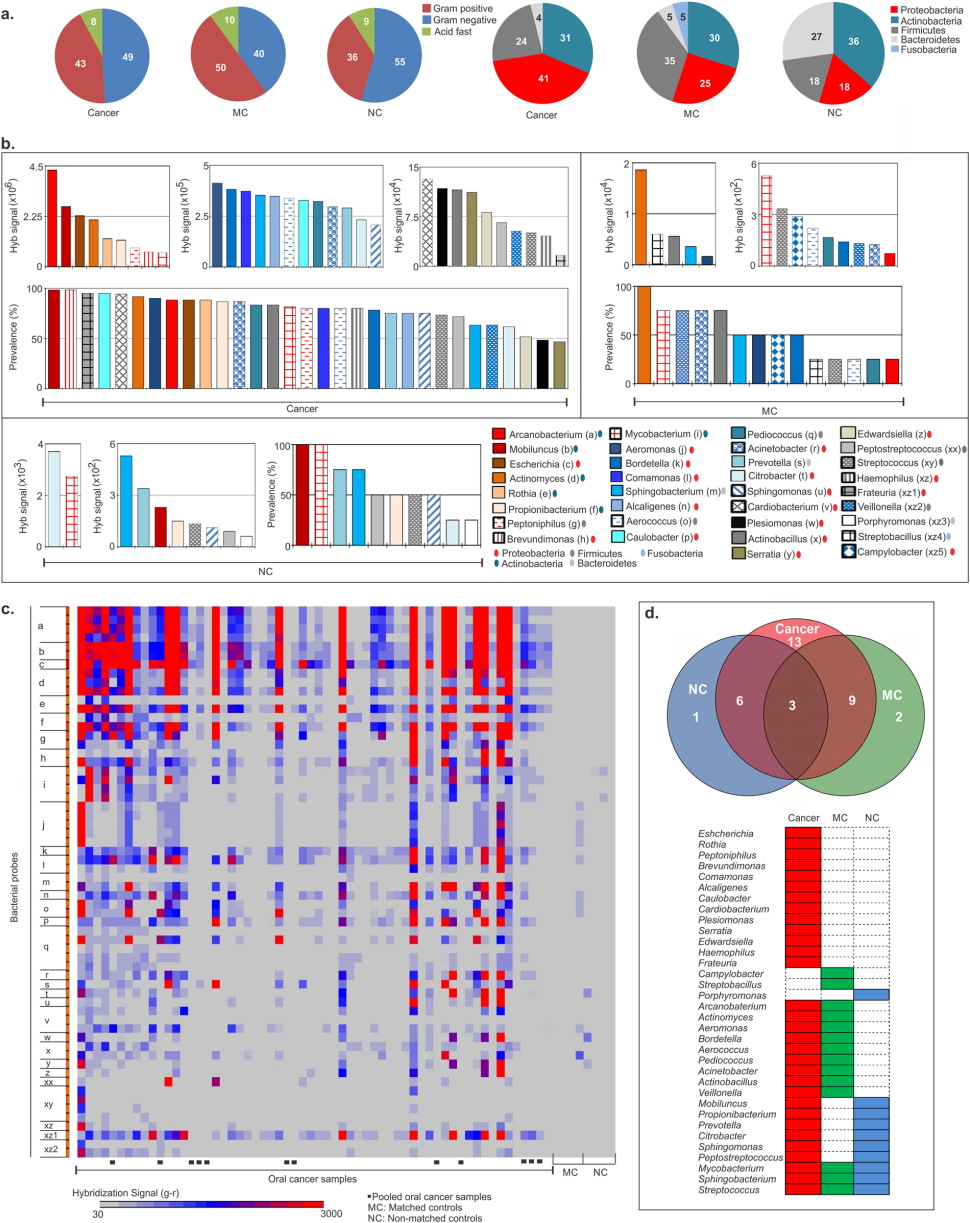
Bacterial signatures detected in oral cancer samples. (**a**) Pie charts showing the percentage of different groups and phyla of bacteria detected in oral cancer, matched (MC) and non-matched controls (NC). (**b**) The bacterial signatures that are detected with hybridization signal (g–r > 30) by PathoChip screen of 100 oral cancer samples and in MCs and NCs are shown and ranked according to decreasing hybridization signal (weighted score sum of all the probes per accession) and prevalence. (**c**) Figure **c** shows the heat map of the hybridization signal for the bacterial probes of bacterial genera a-xyz, labeled in figure **b**, detected by PathoChip screen with the cancer, matched (MC) and non-matched control (NC) samples. Samples were screened individually and in pools (marked ▪). (**d**) Figure **d** shows the association of molecular signatures of different bacterial genera with oral cancer and/or controls, represented as a venn diagram,﻿ and as colored bars.﻿

Figure 2 and Table S2 shows the variety of bacterial signatures found in OCSCC, matched and non-matched control samples. These include Proteobacteria, Actinobacteria, Firmicutes, Bacteroidetes, and Fusobacteria. There were observed differences in gram-positive and gram-negative microbiota in OCSCCs compared to control samples. In the non-matched controls about 55% of the organisms were gram-negative compared to 40% in the matched controls and 49% in the OCSCC samples. 43%, 50% and 36% of the bacterial agents were gram-positive in the OCSCCs, matched and non-matched controls, respectively (Fig. 2a). Interestingly, Proteobacteria, one of the major gram negative phylum (includes *Esherichia*, *Vibrio* and *Salmonella*) was much more pronounced in OCSCCs at 41% compared to matched and non-matched control at 25% and 18%, respectively (Fig. 2a). The Bacteroides were more pronounced in the non-matched controls at 27% compared to 4% and 5% in the OCSCC and matched controls, respectively (Fig. 2a). The gram-positive phylum Actinobacteria was similar across all samples at 31%, 30% and 36% (Fig. 2a). The Firmicutes phylum of gram-positive bacteria was more pronounced in the matched controls at 35% compared to 24% and 18% in OCSCCs and non-matched controls, respectively (Fig. 2a). Among the bacterial signatures detected in the OCSCC samples (Table 1 and Fig. 2b), Proteobacteria *Brevundimonas* and Actinobacteria *Mobiluncus* were the most prevalent (98%) followed by the generas of *Frateuria*, *Caulobacter*, *Actinomyces*, and *Aeromonas*, that were detected in about 90% of the cancer cases. Probes of the Actinobacteria detected in the OCSCC samples had high hybridization signals (Table 1 and Fig. 2b), the highest being that of *Arcanobacterium*. While probes of Proteobacteria generas *Esherichia* and *Brevundimonas* were detected in 88% and 98% of cancer cases, respectively with high hybridization signals (Fig. 2b,c and Table 1), the other Proteobacteria generas detected in cancer cases showed low to moderate hybridization signals, but interestingly, they were highly prevalent (>75%), except for the generas *Serratia*, *Plesiomonas*, *Edwardsiella*, *Citrobacter* (46–62%) (Fig. 2b and c).

As expected, the matched control samples shared some of the bacterial signatures that were detected in the cancer samples along with other bacterial signatures of normal oral flora (Fig. 2b). Table S2 shows the list of bacterial genera detected and shared among the cancer, matched and non-matched control samples. Bacterial signatures of the genera *Actinomyces* were detected with the highest prevalence (100%) and hybridization signal intensity in the matched controls (Fig. 2b). 8 of the 14 bacterial genera detected in matched controls were also detected in the OCSCC samples (Table S2). They represented the genera of *Arcanobaterium*, *Actinomyces*, *Aeromonas*, *Bordetella*, *Aerococcus*, *Pediococcus*, *Acinetobacter*, and *Veillonella* (Table S2). Among the non-matched control samples, bacterial signatures of generas *Mobiluncus* and *Mycobacterium* were detected in all samples, and probes of generas *Citrobacter* and *Mycobacterium* showed high hybridization signal (Fig. 2b). Importantly, it should be noted that the most of the bacterial signatures detected in the control samples are of the normal oral flora.

The Venn diagram (Fig. 2d) and Supplementary Table S2 summarizes our findings showing that bacterial signatures representing 13 genera are found to be specifically associated with OCSCC samples and not with the matched or non-matched controls which included 11 genera of Proteobacteria, 1 genera of each of Actinobacteria and Firmicutes. As in the case of the viruses, the bacterial microbial signatures showed a significant divergence in the OCSCC when compared to the normal signatures and were more robust.

#### C. Fungal signatures associated with OCSCC

**Figure 3:**
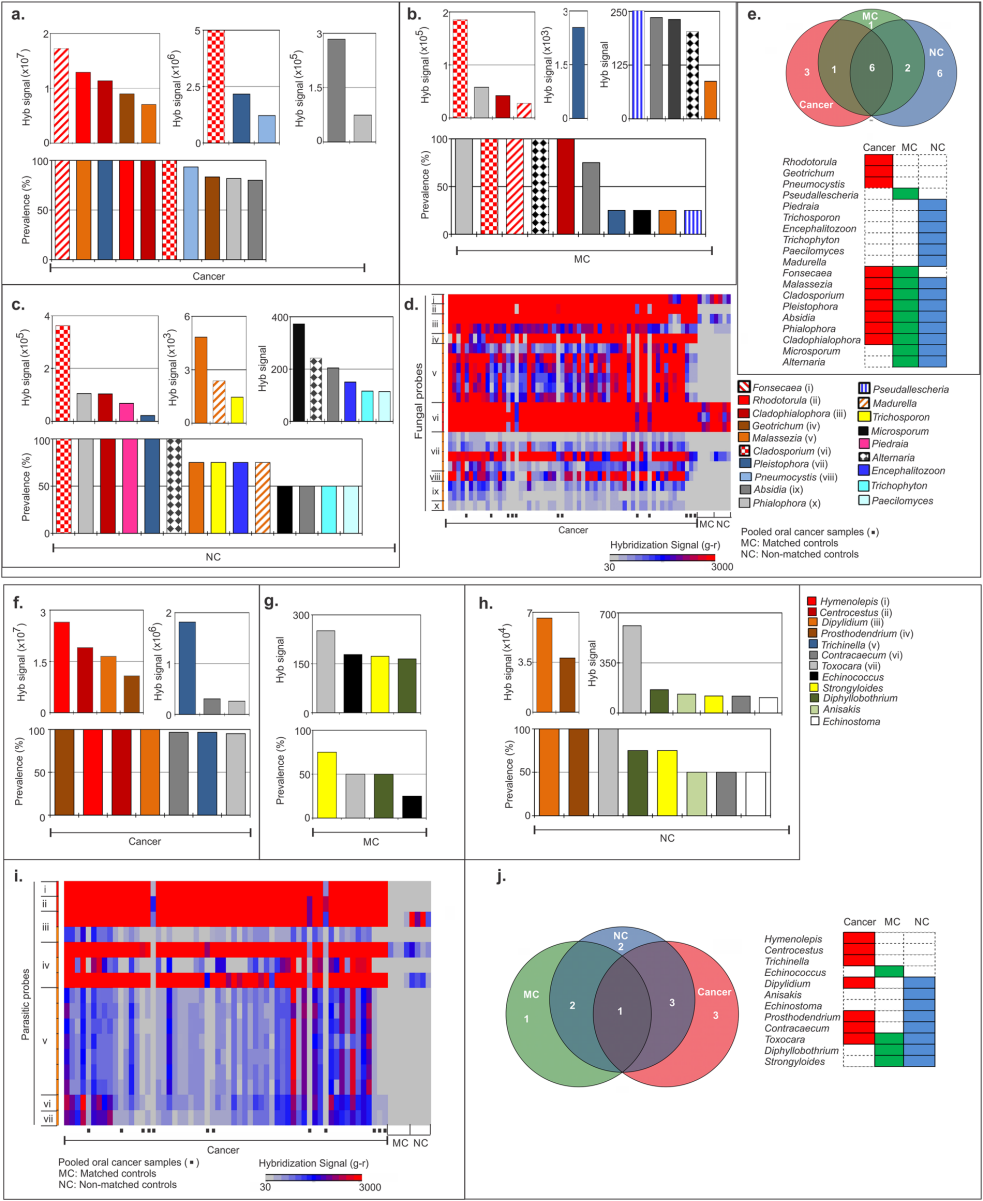
Fungal (**a**–**e**) and parasitic (**f**–**j**) signatures detected in oral cancer samples. (**a**) The fungal signatures that are detected with hybridization signal (g–r > 30) by PathoChip screen of 100 oral cancer samples are shown and ranked according to decreasing hybridization signal (weighted score sum of all the probes per accession) and prevalence. (**b** and **c**) Figure **b** and **c** shows the fungal signatures detected in the matched (MC) and non-matched controls (NC) respectively, ranked according to decreasing hybridization signal and prevalence. Figure **d** shows the heat map of the hybridization signal for the fungal probes of fungi i–x, labeled in figure **a**, detected by PathoChip screen with the cancer, matched (MC) and non-matched control (NC) samples. Samples were screened individually and in pools (marked ▪). (**e**) Figure **e** shows the association of molecular signatures of different fungal genera with oral cancer and/or controls, represented as a venn diagram, and as colored bars. (**f**) The parasitic signatures that are detected with hybridization signal (g–r > 30) by PathoChip screen of 100 oral cancer samples are shown and ranked according to decreasing hybridization signal (weighted score sum of all the probes per accession) and prevalence. (**g** and **h**) Figure **g** and **h** shows the parasitic signatures detected in the matched and non-matched controls (MC and NC) respectively, ranked according to decreasing hybridization signals and prevalence. (**i**) The heat map of the hybridization signal for the parasitic probes of parasites i–vii, labeled in figure **f**, detected by PathoChip screen with the cancer, matched (MC) and non-matched control (NC) samples. Samples were screened individually and in pools (marked ▪). (**j**) Figure **j** shows the association of molecular signatures of different parasitic genera with oral cancer and/or controls, represented as a venn diagram, and as colored bars.

Among the fungal signatures detected in the OCSCC samples were those typically seen in the normal oral flora as well as those that are opportunistic infectious fungi. Molecular signatures of *Fonsecaea*, *Malassezia*, *Pleistophora*, *Rhodotorula*, *Cladophialophora* and *Cladosporium* were detected in all the OCSCC samples screened, *Pneumocystis* was detected in 93% of the cancer samples, and signatures of *Geotrichum*, *Phialophora*, *Absidia* and *Prevotella* were detected in >75% of the cancer cases screened (Fig. 3a). The signatures with high hybridization signal intensity in the OCSCC samples included that of *Fonsecaea*, *Rhodotorula*, *Cladophialophora*, *Geotrichum* and *Malassezia*, with the highest being for *Fonsecaea* (Fig. 3a,d and Table 1).

For the control samples screened, the matched controls detected some of the common oral flora along with some fungal signatures that were detected in the cancer samples (Fig. 3b and e). All the matched control samples significantly detected probes of *Phialophora*, *Cladosporium*, *Fonsecaea*, *Alternaria* and *Cladophialophora* (Fig. 3b), all of which were detected in the matched control samples with high hybridization signal intensity, except for the probes of *Alternaria* (Fig. 3b). Probes of *Absidia* were detected with low hybridization signal intensity in 75% of the matched control samples screened. Among the fungal signatures detected in the non-matched control samples, signatures of *Cladosporium*, *Phialophora*, *Cladophialophora*, *Piedraia*, *Pleistophora* and *Alternaria* were detected in all (Fig. 3c), with high hybridization signals except for the probes of *Alternaria* (Fig. 3c and d). The probes of *Cladosporium*, a common oral flora were detected with the highest hybridization signal intensity in both the matched and non-matched control samples, and were also detected at similar intensity in the cancer samples (p > 0.05) (Fig. 3a–d and Table 1).

The Venn diagram shows the shared and specific fungal signatures between OCSCC, matched and non-matched controls. Noteworthy are the three fungal signatures, *Rhodotorula*, *Geotrichum* and *Pneumocystis*, associated specifically with OCSCCs (Table S2 and Fig. 3e). Again we note that a significant change in the fungal biome of OCSCC was observed when compared to control oral samples.

#### D. Parasitic signatures associated with OCSCC

We detected distinct molecular signatures for parasites in OCSCCs (Fig. 3f,i and Table 1). Probes from 28S and/or 18S rRNA of *Hymenolepis*, *Centrocestus*, *Dipylidium* and *Prosthodendrium* were detected in all the OCSCC samples with very high hybridization signal (Fig. 3f,i and Table 1). Probes of *Contracaecum*, *Trichinella* and *Toxocara* were detected in >95% of the cancer samples with moderate hybridization signal intensity (Fig. 3f,i and Table 1).

Signatures for *Toxocara*, which were detected in OCSCC samples, were also detected in 50% of the matched control samples screened, with lower hybridization signals along with parasitic signatures of *Strongyloides* and *Diphyllobothrium*, which were detected in 75% and 50% of the matched control samples respectively (Fig. 3g and i). In the non-matched control samples we detected *Dipylidium* and *Prosthodendrium* with high hybridization signal intensity in all the samples screened. However, the hybridization signals of both in the non-matched controls were significantly lower than that in the OCSCC samples (Table 1). Parasitic signatures of *Toxocara* were also detected in all the non-matched control samples screened with moderate hybridization signal intensity, along with the probes of *Diphyllobothrium*, and *Strongyloides* detected in 75% of the non-matched controls (Fig. 3h and i).

The Venn diagram in Fig. 3j summarizes the findings of parasitic signature associations with cancer and control samples. Molecular signatures of *Hymenolepis*, *Centrocestus* and *Trichinella* were found to be associated only with OCSCC. Signatures of *Echinococcus* was found to be associated only with matched control samples and that of *Anisakis* and *Echinostoma* was found to be associated only with non-matched control samples. Thus distinct signatures differentiate cancer, matched controls and non-matched controls.

### Hierarchial clustering of OCSCC samples based on detection of microbial signatures

**Figure 4:**
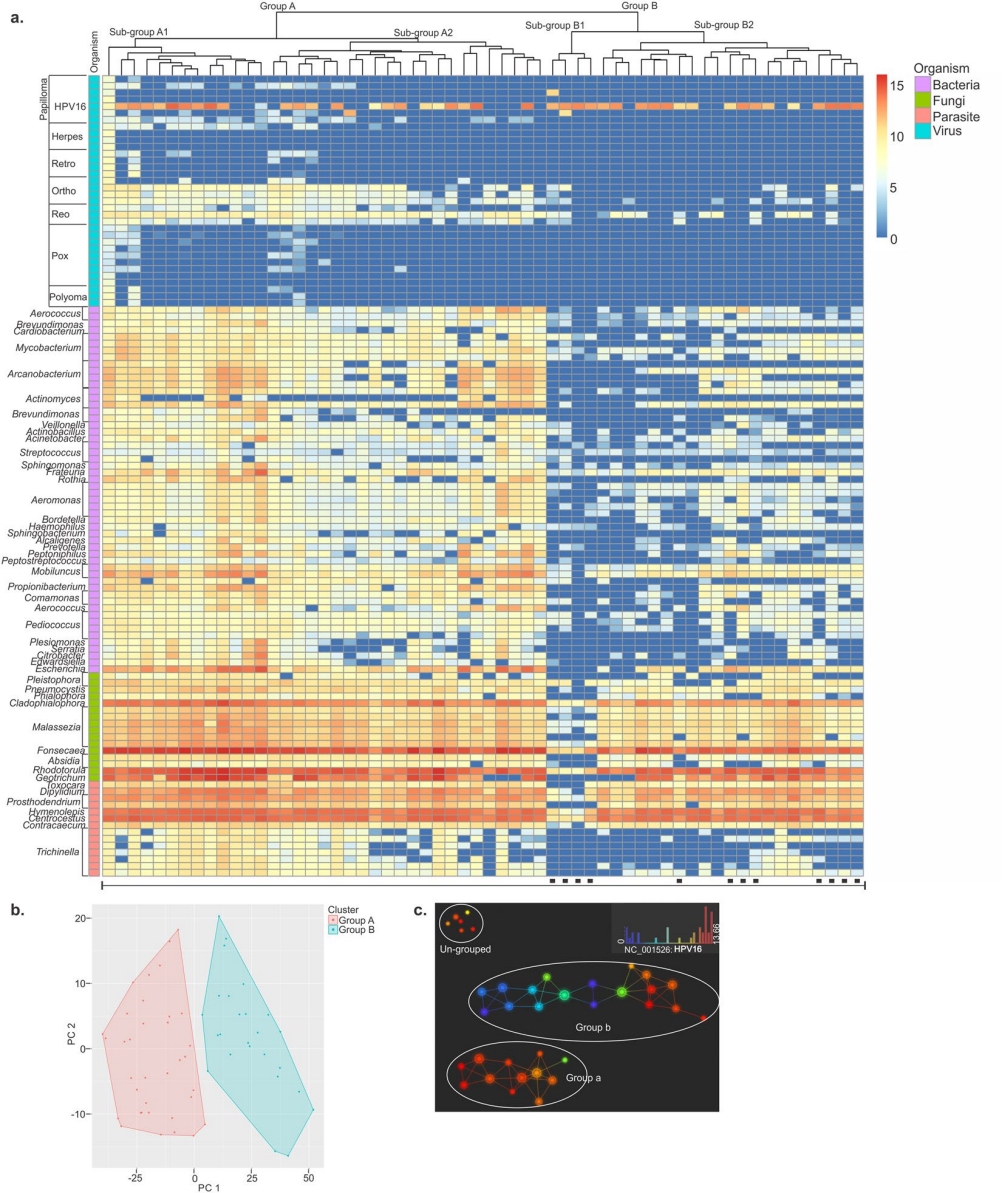
Hierarachial clustering of 100 oral cancer samples. (**a**) Hierarchial clustering by R program using Euclidean distance, complete linkage and non-adjusted values. Samples marked (▪) were the samples that were screened in pools, rest were screened individually. (**b**) Clustering of the OCSCC samples using NBClust software [CH (Calinski and Harabasz) index, Euclidean distance, complete linkage]. (**c**) Topological analysis using Ayasdi software, using Euclidean (L2) metric and L-infinity centrality lenses. The OCSCC samples that had similar detection for viral and microbial signatures formed the nodes, and those nodes are connected by an edge if the corresponding node have detection pattern in common to the first node. Each nodes are color coded according to the detection of HPV 16.

Hierarchial clustering was done based on the detection of the microbial signatures in the 100 OCSCC samples. Signature of *Cladosporium* was ignored as it was not significantly detected in the cancer samples compared to the controls. Hierarchial clustering analysis using the R program showed that the OCSCC samples fell into 2 major groups (A and B) based on specific microbiome (Fig. 4a). Molecular signatures for HPV16 were detected in the 2 major groups identified (Fig. 4a). Apart from HPV16 probes, group A OCSCC samples also showed signatures of other viral probes, primarily belonging to Orthomyxoviridae and Reoviridae (Fig. 4a). The bacterial signatures were broadly detected in group A samples, compared to the sporadic bacterial signatures detected in group B samples, with *Rothia* and *Mobiluncus* detected in both A and B groups. Both group A and B had high detection of some fungal and parasitic signatures except for the parasitic signature *Trichinella* which was either absent or sporadically detected in group B while detected in almost all the group A samples. Thus we observed in group A OCSCC samples, higher detection of viral, bacterial and parasitic signatures of *Trichinella*, compared to group B. Group A samples separated into two subgroups (A1 and A2 which were primarily differentiated by higher hybridization signals for some viral, bacterial and parasitic probes in sub-group A1. Group B also differentiated into two subgroups (B1 and B2) where sub-group B1 had a generally lower level of detection of bacteria compared to sub-group B2. However, both group A and group B OCSCC samples were positive for bacterial signatures of *Frateuria*, *Mobiluncus*, fungal signatures of *Cladophialophora*, *Fonsecaea* and *Rhodotorula*, and all the parasitic signatures except for *Trichinella* in addition to HPV16 signatures.

Clustering of the OCSCC samples were done using NBClust software as described in the methods section (Fig. 4b). We again observed two distinct clusters 1 and 2, similar to the one described above. While there were no significant differences between the two clusters for the signatures of HPV 6b, HPV 16, HPV 26, HHV 8, HHV 6B, HHV 5, retroviral signatures, certain pox viral signatures, parapox viral signatures and polyoma viral signatures, there were significant differences in the detection of some of the viral and all the bacterial, fungal and parasitic signatures between the two clusters, cluster 1 having higher detection than 2 (Table S5). In this cluster analysis some viral signatures were analyzed as significantly more prevalent in cluster 1 than in cluster 2. ﻿These included Orthomyxoviridae, Reoviridae, HPV 34, HHV 6A, Mouse mammary tumor virus-like (MMTV-like) and some poxviruses.

Additional analyses using a topological approach represented data by grouping cases with similar detection for viral and microbial signatures into nodes, and connecting those nodes by an edge if the corresponding nodes have detection pattern in common to the first node (Fig. 4c). Topological analysis visualized all the OCSCC cases into two clusters, ‘Group a’ and ‘Group b’, along with some cases that did not have common detection pattern (ungrouped or singletons) (Fig. 4c). The nodes were colored based on the HPV16 hybridization signal intensity in the samples. The color of the nodes from blue to red represented the samples with no to high detection for HPV16. Groups a and b showed significant differences in detection of certain micro-organisms which are listed in Table S6. Importantly, there was significantly higher detection of HPV16 in ‘Group a’ compared to ‘Group b’. The samples within ‘Group b’ ranged from having no to very high HPV16 signals as noted by the greater number of red nodes (Fig. 4c). The 6 un-grouped samples had significantly lower detection of the majority of microbial signatures that were detected in the ‘grouped’ samples [Table S6, un-grouped vs group (a + b)], except for fungal signatures of *Cladosporium* and viral signatures of Polyomaviridae and HPV16.

The clustering analysis clearly showed that the OCSCC samples fall into at least two distinct microbial signatures. Further study and understanding of the different signatures may provide diagnostic and prognostic capabilities.

### Validation of PathoChip results of OCSCC by probe capture and next generation sequencing

To verify the PathoChip results we chose conserved and sequence specific probes for a number of viruses, bacteria, fungi and parasites that had positive detection in the PathoChip screen. These were conjugated with biotin, and streptavidin beads were used to capture the biotinylated probe-DNA/cDNA complexes from the amplified genomic DNA/cDNA pool of the OCSCC samples. The resulting enriched targets were subjected to MiSeq, and the sequence reads were aligned to the PathoChip metagenome^20^.

**Figure 5:**
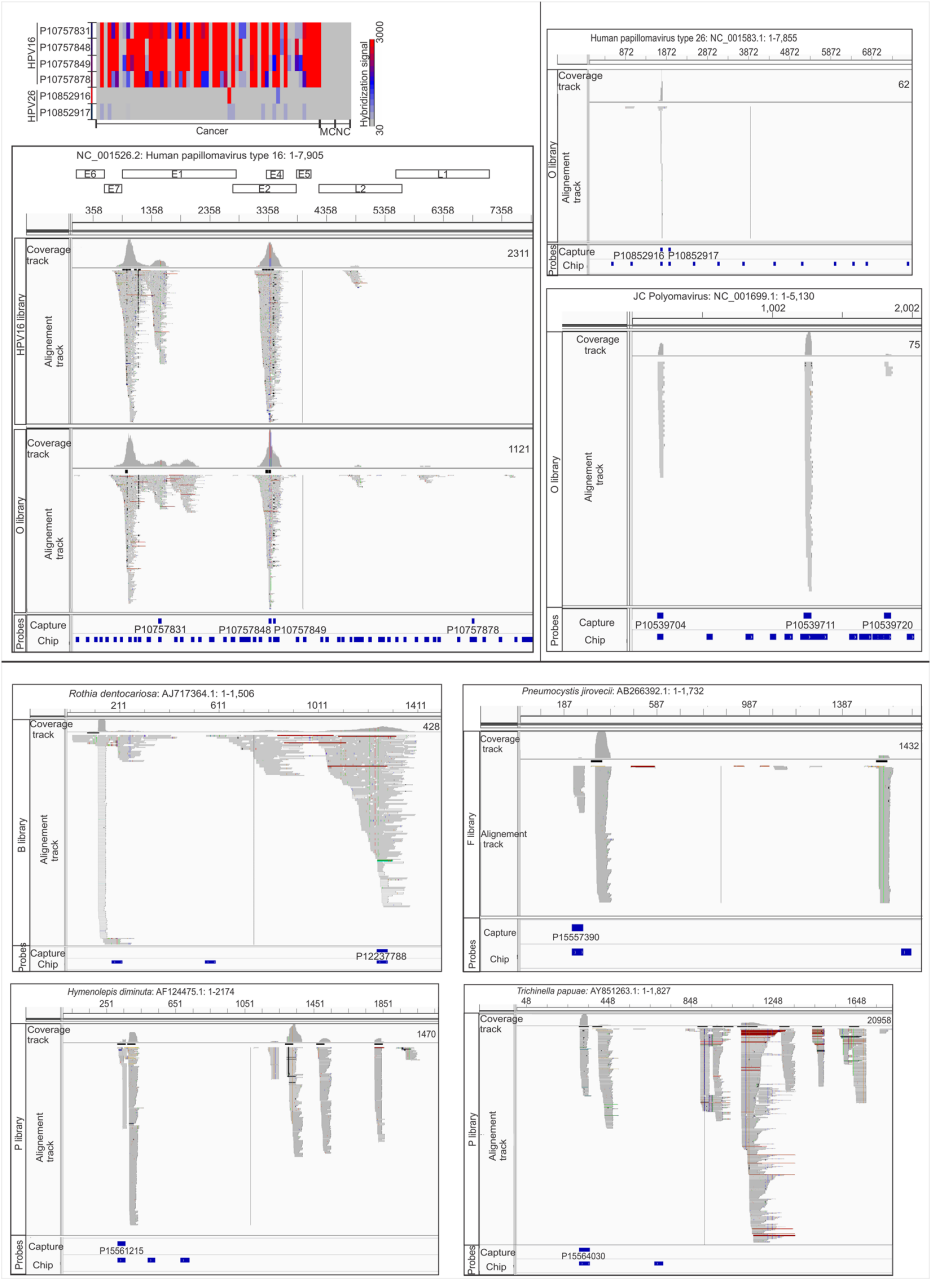
Probe capture sequencing alignment is shown for individual capture pools (HPV16, O, B, F and P). HPV16 capture probe comprised of set of HPV16 specific probes, O capture probes consisted of certain viral and bacterial probes, B pool comprised of bacterial probes, F consisted of fungal probes and P comprised of parasitic probes that are mentioned in Table S3. The hybridization signals of the HPV probes used for the capture are shown as heat map in the figure. Six pools of whole genome and transcriptome amplified DNA plus cDNA was hybridized to a set of biotinylated conserved and specific viral probes, then captured on streptavidin beads, and used for tagmentation library preparation and deep sequencing with paired –end 250-nt reads. The miseq reads from individual capture when aligned with the metagenome of PathoChip (Chip probes) was found to cluster mostly at the capture probe regions. The genomic location along with the number of miSeq reads are mentioned in the figure for each organism.

The results showed that the sequence reads clustered around the genomic locations of the probes (Table S4 and Figs 5 and S1a–f). However, regions of the target genome outside the capture probe locations were also detected (eg. sequence reads of *Trichinella papuae*, Fig. 5). Four HPV16 specific capture probes from the E1, E2/E4 and L1 genes, used in the reaction pulled out genomic sequence of HPV16 that aligned with most of the HPV16 E1, E2/E4, L2 and L1 genes (Fig. 5). The conserved probe for Polyomavirus from the regulatory region (182–226 bp of NC_001699.1) and specific probes from the late mRNA as well as VP2/VP3 and VP1 region of the JC Polyomavirus were used to enrich JC Polyomavirus genomic regions. All of the captured sequences of JC Polyomavirus were found to align at the genomic regions of the capture probes (Fig. 5). Capture probes designed from 16S rRNA region of the bacteria *Rothia*, captured most of the genomic sequence of the bacteria. Thus the sequence reads aligned not only with the capture probe region, but also extended across the genome of the bacteria (Fig. 5). Other bacterial sequence reads aligned with their respective capture probe regions, further validating the PathoChip screen results (Figure S1c and d). Sequence reads of fungi were also found to align with sequences at or adjacent to their respective capture probe regions (Figs 5 and S1e). For example, 1432 sequence reads of *Pneumocystis*, aligned at the capture probe location in their genome as well as outside of it (Fig. 5). 2057 sequence reads of *Pleistophora*, aligned at the capture probe location of its genome (Figure S1e). High sequence reads (>4000) were obtained for the skin fungus *Malassezia* (Figure S1e).

Sequence alignments of the reads of parasites *Hymenolepis* and *Trichinella* also extended beyond their respective capture probe locations, thus further confirming the presence of nucleic acids from these micro-organisms in the cancer samples. The number of reads were extremely high for *Trichinella* (>20,000) and *Prosthodendrium* (>9000) (Figs 5 and S1f,g), suggesting higher capture of the genomic signatures of these two micro-organisms in OCSCC.

These capture and sequencing approach validated the presence of genomic regions of the viruses and microbes detected by the PathoChip analysis, confirming their presence in the OCSCC samples. Although we did not test every virus or microbe detected we found that all those tested were strongly verified, suggesting that the overall signatures detected are valid.

### Insertions of a broad range of microbial genomic fragments were identified in the host human chromosomes

**Figure 6:**
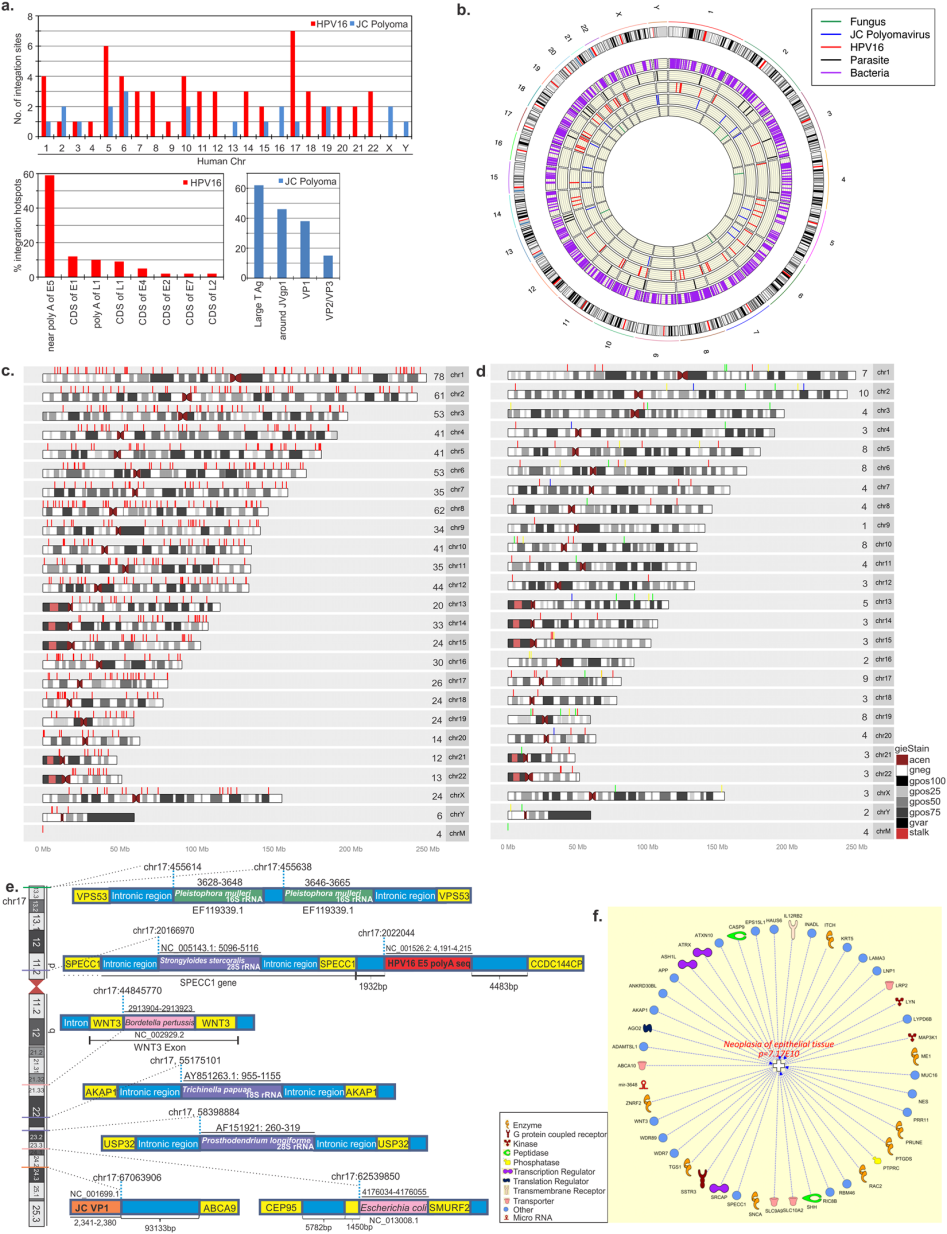
Microbial genomic integrations in the host chromosome. (**a**) Bar graphs showing number of viral (HPV16 and JC Polyomaviral) integration sites in host human chromosomes and the percentage of viral genomic sites for integration into host chromosomes. (**b**) Circos plot highlighting fusion events with >=20 reads support for the bacterial, fungal and parasitic insertions into individual human chromosomes are shown. For the viral insertions, all the reads were taken into account. (**c**) Karyogram plot of bacterial insertion sites (red lines) in human chromosomes, cut off reads >=20. The number of insertion sites in each chromosome is mentioned in the figure before chromosome number. (**d**) Karyogram plot of virus, parasite, fungus, insertion sites in human chromosomes. Color profile: green lines for parasite genomic insertional sites, red for HPV16, yellow for JC Polyomavirus, blue for fungus. The cutoff read for bacteria, fungus and parasite, >=20 and for virus, all the insertion sites were included. The number of insertion sites in each chromosome is mentioned in the figure before chromosome number. G-banding annotation for each chromosome is shown; gneg - Giemsa negative bands; The Giemsa positive bands have further been subdivided into gpos25, gpos50, gpos75, and gpos100 with the higher number indicating a darker stain; acen - centromeric regions; gvar - variable length heterochromatic regions; stalk - tightly constricted regions on the short arms of the acrocentric chromosomes (**e**) Schematic representation of viral and microbial genomic insertional sites in human chromosome 17. The genomic co-ordinates of the pathogens integrated and that of the host chromosome integration sites are mentioned. The co-ordinates for human chromosomes are from GRCh37/hf19 Assembly. (**f**) Association of host genes affected by viral/microbial genomic integrations to neoplasia of epithelial cells, analysed by Ingenuity Pathway Analysis (IPA) program that showed a *p*-value of 7.17E10 for such association.

An important question arising from our data is whether or not the viral or microbial signatures detected may result from elements integrated into the human genomes. Thus we analyzed the captured sequences for regions that partly align to the microbial genomic regions and partly to human sequence, using VirusClip method that would suggest integration. The analysis detected numerous viral and microbial genomic insertional sites within the human chromosomes (Fig. 6). We identified 38,019 bacterial insertional sites, 125 fungal genomic insertional sites, 508 parasitic insertional sites and 79 viral insertional sites (Fig. 6b–d and Tables S7–S10). To simplify the data we focused on reads > 20 for bacterial, fungal and parasitic sequence fusion with host genome. Figure 6b represents the data in a Circos plot highlighting the insertions. Although the numbers of viral insertions were lower compared to other microbial insertions, we included the 79 insertional sites for JC and HPV16. The Circos plot shows insertions going from the inner concentric circle to outer circle in the order of fungus, JC Polyomavirus, HPV16, parasites and bacteria. This is then comprehensively shown with its represented colors in the outermost circle with all insertions (Fig. 6b). A karyotype plot also shows the representative bacterial and fungal, parasitic and viral insertional sites in each chromosome (Fig. 6c and d). The number of insertions for each chromosome is shown to the left of each chromosome number. Bacterial insertions are shown for all chromosomes in Fig. 6c. Interestingly, chromosomes 1, 2, 3, 6 and 8 showed over 50 bacterial genomic insertions each, and the Y chromosome having the least number of bacterial genomic insertions (Fig. 6c). Notably, the mitochondrial chromosome also showed 4 bacterial genomic insertions in our analysis (Fig. 6c). Insertions for viral, fungal and parasitic agents although less frequent were seen in all chromosomes (Fig. 6d). While chromosomes 2, 5, 6, 10, 17 and 19 had more such insertions, chromosome 9 only had 1 such insertion (Fig. 6d). Interestingly, the sites for microbial insertions were exonic, intronic, upstream/downstream and at the UTRs or at the ncRNA region of host genes.

These data, based on a very sensitive analytical approach, suggest that there is a far greater intimacy between human and microbial genomes, at the level of integration, than previously observed.

#### A. Viral integrations into host genomes

Genomic elements of HPV16 and JC Polyomavirus were found to be integrated in the chromosomes of OCSCC cells. For HPV16, we detected 7 insertion sites in chromosome 17 (chr17), 6 in chromosome 5 and between 1 and 4 sites in all other chromosomes except chromosomes 13, X and Y (Fig. 6a). The genomic fragment of HPV16 that was identified most frequently integrated in the human genome (59%) was at the genomic co-ordinates 4,172 (based on accession NC_001526.2^21^), which is located prior the polyA sequence of the E5 gene^21^. Additionally, 12% of the HPV16 integrations included HPV genomic co-ordinates 3,393–3,425 in coding sequence of the E1 gene; 10% at co-ordinates 7,206–7,627 near the polyA sequence of the L1 gene), 9% in the coding region of L1 from co-ordinates 6,030–6,715; as well as lower percentage integrations in the coding sequences of the E4 gene (3,358–3,394), the E2 gene (3,393–3,425), the E7 gene (674–693) and the L2 gene (5,201–5,221)^21^ (Fig. 6a).

Figure 6e and Tables 2 and S7 and Figure S2 outline the many HPV16 viral integration sites in the human genome. Most of these were in intronic regions. It is clear that among these many insertions some could disrupt gene expression in ways that could promote oncogenesis.

**Table 2:** Microbial genomic integration sites in the OCSCC host somatic chromosomes.

Microbial insertion region	Human genomic Integration sites
**HPV16 insertions**	
HPV16 4, 188–4, 243 (hotspot for integration)	Intronic (53% integrations) regions of LAMA3, ATXN10, INADL, ABCA10, EVC2, WDR89, CADPS2, HAUS6, EPHA6, FAM179B, COL14A1, MRPS27, FUCA2, ADAMTS12, TRIOBP, CSMD1, KCNQ1.
	Upstream (12%) of genes IL12RB2, LOC388436, LOC79999, FCHO1, MRPL52, SLC7A7
	Downstream (9%) of the genes NACAP1, GUCA2A/GUCA2B, RSPH1
	Intronic ncRNA gene of the FAM35BP gene (6% integrations)
	Intergenic integrations (6%):
	−upstream of SPECC1; downstream of CCDC144CP
	−upstream of SSTR3 and downstream of RAC2.
HPV16 E1	Intronic region of SLC13A3, DLGAP1, CCDC155 and ncRNA LOC10028863
	Intergenic regions
HPV16 E2 and E4	Intronic region of LOC10272495
	Intergenic regions
HPV 16 L1	Intronic region of PAFAH1B1, ncRNA LOC10050620
	Intergenic region
L1 PolyA	Intronic regions of DEPDC4
	3′ UTR region of the MKLN1
	Intergenic regions
HPV 16 L2	Intronic region of SSH2
**JC Polyomavirus insertions**	
JC LT Ag	Intronic regions of CMTR1 and ME1 on chr6; CPO on chr2
	Intergenic regions of chromosomes 1, 2 and 3
VP1 ORF	Intergenic regions, 41 Kb downstream of the lncRNA gene SFTA1P (chr10)
	Upstream of ABCA9 (chr17)
	3′ UTR of the epigenetic regulator gene MECP2
VP2 and VP3	Intronic region FAM13B (chr 5) and PCCA (chr 13)
Agnoprotein Jvgp1	Intronic regions MSH3 (chr 5) and PHLSB3 (chr19)
Late coding region (191–253)	Intergenic regions of chromosome 16
	−97 Kb downstream of NPIPA7
	−99 Kb upstream of the NPIPA5 gene
	Intronic region of SSG5 (chr15)
**Bacterial insertions**	
*Mycobacterium*	Exon of ADAMTSL1 (chr9)
	Intron of MAP3K1
*Aeromonas*	Exon of RASSF5 (1q32.1)
	3′ UTR of SEC14L4
*Sphingomonas*	Exon of SRCAP (chr16)
	Exon of WNT3 (chr17)
	5′ UTR of C1orf162
	Intron of SLC9A9
*Escherichia coli*	3′-end of SMURF2
	Intron of CASP9
*Campylobacter*	3′ UTR of COL1A1
*Brevundimonas*	Itron of RIC8B
*Pediococcus*	Intron of LYPD6B.
**Fungal insertions**	
*Geotrichum*	Intergenic- 560 Kb upstream of the GABRG1 gene (chr4)
*Pleistophora*	Intron of ITCH (chr20)
	Intron of MAGI1 (chr 3)
*Phialophora*	Intron of ZNRF2 (chr7)
*Rhodotorula*	Intron of CADPS2 (chr7)
**Parasitic insertions**	
*Strongyloides*	Exon of ZNF383 (chr19)
	Intron of LNP1 (chr3)
	Intergenic- downstream of SLC10A2 (chr13)
	Intron of SPECC1 (chr17)
*Contracaecum*	Exon of RHD (chr1)
*Trichinella*	intron of AKAP1 (chr17)
	Intron of EPS15L1 (chr19)
	Intergenic- 353 Kb upstream of NRG3 (chr10)
*Echinococcus*	Intron of ATRX (chrX)
	21 Kb upstream of FGFR2
*Prosthodendrium*	Intron of USP32 (chr17)
	Intergenic region- 37 Kb upstream of Lyn gene
*Hymenolepis*	Downstream of MIR3648 (chr21)
*Diphyllobothrium*	Intergenic- 106 Kb upstream of TRIM49B (chr9)
	in the ncRNA ANKRD30BL gene

JC Polyoma (JC) viral genomic integration was observed in human chromosomes 1, 2, 5, 6, 10, 13, 15, 16, 17, 19, X and Y (Fig. 6a). The JC viral (accession NC_001699) genomic integrations were mostly (38%) within the large T antigen gene (co-ordinates 2,623–2,653)^22^. Additionally, 14% of integrations involved regions around the JVgp1 gene (191–460), 24% in the VP1 gene (1,479–2,361) and 10% in the VP2/VP3 gene (1,278–1,318) (Fig. 6a).

Tables 2 and S7 show the various integration sites for JCV in the human genome, again these insertions could affect gene expression in ways that would promote oncogenesis.

#### B. Integration of bacterial genomic elements in host chromosomes

We observed insertional sites for bacterial genomic fragments in exonic, intronic, intergenic, 3′ and 5′ UTR region, upstream and downstream regions of numerous genes of human chromosomes (Fig. 6 and Table S8). We detected at least 890 bacterial sequence insertional sites at different exons of human chromosomes, listed in Table S8. Several particularly interesting inserts within human gene related to cancer are shown in Table 2. We detected *Mycobacterium* (NC_008595.1) genomic elements 24065–24105 insertions at the exonic regions of the tumor suppressor ADAMTSL1 gene on chromosome 9; *Aeromonas* (NC_008570.1) genomic elements insertion sites in the exon of the RASSF5 (1q32.1), a member of Ras association domain family that functions as a tumor suppressor and shown to be inactivated in a variety of cancers^23^; *Sphingomonas* (NC_009511.1) genomic elements insertions in exonic regions of chromatin re-modelling gene SRCAP on chromosome 16; Bordetella (NC_002929.2) genomic insertional site within the exon of the proto-oncogene WNT3 on chromosome 17 (Fig. 6); Escherichia coli (NC_013008.1) genomic insertional site at the end of the SMURF2 gene, a tumor suppressor and regulator of the G1/S checkpoint^24, 25^.

Apart from the exonic insertional sites, we also detected numerous sites (514) at the 3′ and 5′ UTR regions of genes. For example, *Bordetella* genomic insertions were seen at the 5’UTR of C1orf162, *Aeromonas* insertions at the 3′ UTR of the SEC14L4 gene; and *Campylobacter* insertions at the 3′ UTR of the COL1A1 gene.

#### C. Integration of fungal DNA elements in host chromosomes

Genomic fragments of the fungal OCSCC flora were also detected at 125 insertion sites in the intergenic (46%), intronic (42%), upstream or downstream of genes or, ncRNA but not exonic regions in the human chromosomes (Fig. 6b–e and Table S9). We listed some of the important fungal genomic integrations in Table 2. The 18S rRNA fungal genomic fragments at the intronic regions of tumor suppressor MAGI1^26^, the negative regulator of tumor suppressor, ITCH gene (Fig. S2) and the E3 ubiquitin protein ligase ZNRF2 (Table S9) are of special mention.

#### D. Genomic insertions of parasitic DNA in host chromosomes

Numerous genomic insertional sites for parasites were detected in the OCSCC cell genomes (Fig. 6d and Table S10). The majority of these insertions were at intergenic (202 sites) or intronic (198) regions. Insertional sites were also detected upstream and downstream, at the splice site, 3 and 5′ UTR regions of certain genes, while only 2 insertional sites were detected in the exons (Table S10). Table 2 highlights some of the integrations that may affect human genes involved in cancer.

The insertional data suggest that there may be far more integrations of viral and other microorganisms than previously expected, and IPA analysis of some of the affected host genes showed that they have a significant association with oncogenesis (*p*-value = 7.17E-10) (Fig. 6f).

## Discussions

Using our pan-pathogen array technology we have defined microbiome signatures specific to OCSCC. The presence of these viruses and microorganisms raise the possibility that they may be involved in initiating, promoting or modulating the cancer. Equally possible is that some of the microbiome members find the tumor microenvironment supportive of their persistence. In either case the OCSCC specific microbiome signature is a potential biomarker for diagnosis and prognosis.

An oncogenic virus, HPV16 was the most detected among the molecular signatures, having the highest average hybridization signal and found in 98% of the OCSCC/OPSCC samples. Previous studies have suggested a 35% prevalence of HPV16^27, 28^. This difference may reflect the fact that our samples came from a surgical practice that focuses on trans-oral robotic resection of OCSCC that are most often associated with HPV 16^29–32^. However, other HPVs (HPV2, HPV6b, HPV1, HPV18, HPV26, HPV34) were detected less commonly, which is in concordance with previous reports^28^. Additional viral signatures detected in OCSCC including Herpesviridae, Poxviridae, Retroviridae and Polyomaviridae; these were dramatically under-represented in the non-matched healthy controls. These observations are of significance because there are no detailed reports of the viral association with OCSCC other than HPVs and herpesviruses^33^. Epstein-Barr Virus (EBV) has been detected in 60% of OCSCC samples in one study^34^, 38% of OCSCC patients in another^35^, and in the majority of OCSCC samples in another study^36^. It has also been suggested that the higher percentage of EBV positivity correlated with the increasing grade of OCSCC^37^. This again suggests that knowledge of the OCSCC microbiome may be diagnostic or prognostic.

Extensive studies have been carried out to look for bacterial flora associated with OCSCC^8, 9, 38–40^. A recent study, showed that the bacterial microbiome over the OCSCC tumors in 13 patients had significant reduction in the abundance of Firmicutes (*Streptococcus*) and Actinobacteria (*Rothia*), and an increase in abundance of Fusobacteria (Fusobacterium), when compared with their respective matched-controls^38^. However, they did not find this trend when the bacterial microbiome was compared between cancer and non-matched controls. In fact, there were a greater abundance of Bacteroidetes (*Prevotella*) in OCSCC patients compared to healthy non-matched controls. Our results on the other hand showed a slight decrease in the abundance of Firmicutes and not much change in the detection of Actinobacteria in oral cancer samples compared to matched controls, whereas, we observed a drastic reduction in the abundance of Bacteroidetes in both cancer and matched controls when compared to non-matched controls. In the present study a significant bacterial signature specific to OCSCC was the increased detection of Proteobacteria, observed in the cancers far more than matched and non-matched controls. Specifically, for the bacteria detected only in the cancer samples (not in the controls), 11/13 belong to Proteobacteria. Of these 11, *Escherichia* and *Brevundimonas* were reported earlier as associated with different cancers^41, 42^. Also specific to OCSCC was the actinobacteria genus *Rothia*, which was previously shown to be associated with cancer in other studies^43^.

In one study, although there were limited differences found when comparing the detection of bacterial flora at the phylum level between cancer and controls, there were significant differences in the bacterial genuses detected within the phylum^40^. The bacterial genuses associated with OCSCC in that study included *Veillonella*, *Fusobacterium*, *Prevotella*, *Porphyromonas*, *Actinomyces*, *Clostridium*, *Haemophilus*, *Enterobacteriaceae*, and *Streptococcus*, most of which were also detected in our OCSCC samples, except for *Clostridium* and *Porphyromonas* ^40^. Interestingly, in our study there were specific bacteria that were in controls but excluded from OCSCC, for example *Porphyromonas* was detected only in the non-matched healthy controls; and *Fusobacterium* was detected with very low hybridization signal (below our cut-off for inclusion) in less than 20% of the cancer samples screened. However, *Fusobacterium* has been detected in other screens and cancers suggesting that there may be differences in the signatures predominant in the oropharynx compared to previously screened oral tissues^40, 44^. Like the present study, one study detected Firmicutes and Bacteroidetes in OCSCC patients^39^. In a separate study the same group identified species of *Streptococcus* like the present study, along with *Gemella*, *Johnsonella* to be associated with the OCSCC tumor site and not with matched controls^9^. Another study significantly detected DNAs of *Prevotella* and *Streptococcus* similar to our study along wih *Capnocytophaga* in the saliva of OCSCC patients versus non-matched control groups and suggested they may be diagnostic indicators of OCSCC^7^. However, we detected species of *Streptomyces* in the OCSCC tumor site, and in both matched and non-matched controls and signatures of *Prevotella* in both OCSCC and non-matched controls.

There are few studies showing an association between fungi and cancer. *Candida* infection has been associated with oral leucoplakias, and studies have shown that such infection had higher rate of malignant transformation^45–48^. We found yeasts like *Rhodotorula*, *Geotrichum* and *Pneumocystis* to be significantly associated with OCSCC/OPSCC tumor, and not with the adjacent matched tissue control samples or healthy non-matched controls. Previous studies have detected yeast like *Rhodotorula* in oral cancer patients^49, 50^. and there have been reports of association of *Pneumocystis* with different cancers^51^. These fungi are well-known opportunistic pathogens, and would likely find the cancer microenvironment amiable for survival. This can also transform harmless commensals to pathogenic oral mucosal micro-organisms, leading to increased morbidity and mortality in cancer patients.

We detected *Fonsecaea* in both OCSCC/OPSCC cancer and the adjacent normal matched control tissues, but not from non-matched controls. This is likely due to spread from the tumor site to the adjacent non-cancerous tissues or the sharing of a common microenvironment that supports *Fonsecaea*. In support of our data, a recent report observed that chronic fungal infection, mainly by *Fonsecaea* species, contributed to OCSCC^52^. Similar to other studies, that detected microspoiridia in cancer patients^53^, we also detected microsporidia *Pleistophora* much more significantly in OCSCC compared to the controls. We also recognized fungi of low pathogenicity like *Malassezia* and *Absidia*, along with the dermatatious aetiologic agents of chromoblastomycosis, *Phialophora* and *Cladophialophora*, associated significantly with the oral cancer patients as compared to both controls. These fungi can cause significant infection and morbidity in cancer patients^54^. The fungi that were detected only in the controls and not in the cancer samples were common dermatatious, low pathogenic fungi.

Some parasitic worms of the human body, as well as parasites acquired by ingesting raw fish and meat can increase the risk of developing certain cancers. We detected molecular signatures of the intestinal parasites, *Hymenolepis*, *Centrocestus* and *Trichinella* in almost all the OCSCC/OPSCC samples screened but not in the control samples. There have been reports on the association of intestinal parasites with different cancers^55–57^.

A general overview of our data shows that we detected an association of certain viral and other microbial signatures with OCSCC (Table S2). We suggest that these be considered potential signatures for oral cancer. The microbial signatures that were associated with cancer as well as adjacent matched control tissues may also be considered as potential biomarkers (Table S2), given there is a possibility of the spread from cancer cells to the adjacent non-cancer cells due to a shared characteristic of the microenvironment.

The above findings would remain speculative if they were not verified by other techniques. We used a probe-capture next generation sequencing to further validate our PathoChip screen results. In this approach we used probes that detected microorganisms in the PathoChip Screen to capture the genomic regions of microbial signatures detected in OCSCC (Figs 5 and S1a–f), and we were able to verify the presence of the selected organisms.

The nature of our capture-sequencing analysis allowed us to assess the potential integration of viruses and microorganisms into host cell DNA. Possibly the most intriguing data of the study is the detection of multiple integration sites for viral, bacterial, fungal and parasitic sequences in the host genome, providing the potential for significant alteration in host gene expressions. We have identified several host genes and genomic regions as the integration sites and, as indicated in the text, some of these are cancer-associated. There have been studies showing distribution of integration sites for HPV16 in host chromosomes and their direct involvement in regulating cellular cancer-related genes^58^. The insertional sites for HPV16 were found mostly in intronic regions, consistent with earlier reports^59, 60^. The highest number of integration sites for HPV16 in our study were seen in chr 17, followed by chr 5, both of which have been reported earlier with multiple integration sites for HPV16^58, 59, 61^. Although intronic and intergenic, the HPV16 and JC Polyomavirus integrations may still alter host gene expressions^58, 62^.

Our data also confirm the previously defined hotspot for HPV16 integration, the region around the polyA sequence of the E5 gene^58^; however our data go further and detected other prevalent integration sites in the HPV16 genome. HPV16 genomic integration sites in the human genome were detected at the intronic/upstream/downstream region of certain genes associated with cancer. For example: the LAMA3 gene, splice variant of which are known to be involved in tumor cell invasion and progression in head and neck squamous cell carcinoma^63^; the ATXN10, whose gene product is a downstream effector of the p53-p21 and p16-pRB tumor suppressor pathways^64^; the IL12RB2 gene, whose expression is known to be upregulated in OCSCC^65^; the cell polarity regulator gene INADL, de-regulation of which has been associated with cancer^66^; the ABCA10 gene, known to be downregulated in many cancers^67^; the WDR89 gene, which is seen to be associated with many cancers; the EPHA6, known to be associated with prostate cancer progression^68^; the ncRNA FAM35BP^69^ and LOC100506207^70^, insertion at the vicinity of these ncRNA region may influence their expression as has been reported for several HPV16 integration sites in the vicinity of numerous miRNAs^58^; the tumor suppressor SPECC1 and IL12RB2 mutations are associated with epithelial cancers^71^; the amino acid transporter gene SLC7A7, dysregulation of which is associated with multiple cancers^72^; the pro-apoptotic tumor suppressor SSTR3 gene^73^; the RAC2 gene, linked to different cancers including head and neck cancer^74^; the SLC13A3 gene associated with enhanced metastasis^75^; the DLGAP1 gene, shown to be associated with OCSCC^76^; the PAFAH1B1 gene, a potential oncogene in lung cancer^77^ and the oncogene MKLN1 associated with different cancers^78^. Therefore we have detected distribution of HPV16 integration sites throughout the genome, many of which have the potential to functionally alter critical cellular gene expression through integration.

We also detected JC Polyomavirus Large T antigen sequence insertions that may lead to transformations by expression of large T antigen, large T antigen-cell gene fusion variants, or by dysregulating the target genes^79^. In particular we detected insertion of the large T antigen sequence in the intron of the ME1 gene, whose de-regulation is associated with numerous cancers^80^. We have also detected JC Polyomavirus VP1, VP2 and VP3 viral genomic sequence insertion sites in multiple regions (intergenic/upstream/downstream) of host chromosomes.

Although viral DNA integration in the human genome is known, little is known about bacterial DNA integrations. A recent study showed that bacterial DNA integrates into host genomes through RNA intermediates and this occurs more frequently in tumors than in normal samples^81^. A consequence could be the alteration of host gene expression, which ultimately, may play a role in carcinogenesis^81^. This previous study detected random integration of *Acinetobacter* DNA in the human mitochondrial genome, and *Pseudomonas* DNA integration in the 5′ and 3′ UTR of 4 proto-oncogenes that showed increased transcription along with its conversion to oncogene^81^. In the present study, we detected numerous bacterial insertion sites, especially in the exons of host genes (Tables 2 and S8) like the tumor suppressors ADAMTS1 (with *Mycobacterium* genomic element integrations), RASSF5^23^ (with *Aeromonas* genomic insertions), the SMURF2 gene^24^ (with *Escherichia coli* genomic insertions), the chromatin re-modelling gene SRCAP (with *Sphingomonas* genomic insertions), the proto-oncogene WNT3 (with *Bordetella* genomic insertions). Hence, bacterial DNA insertions in the exonic regions of those genes may alter their expression, suggesting a role in driving oncogenesis. Apart from exonic insertions of bacterial DNA, we also detected numerous insertional sites at the intronic, UTR, ncRNA, and upstream and downstream of host genes involved in many cellular functions that can contribute to neoplasia (Tables 2 and S8).

No previous reports are available for fungal genomic integrations and only one study suggested that sequences of the parasite *Trypanosoma cruzi* were integrated into human somatic cell genomes, disrupting host genes^82^. In the present study, the fungal genomic sequence insertions in the host genome were mostly intergenic or intronic. We found parasitic sequence insertions in the proximity of proto-oncogenes, tumor suppressors and miRNAs which may alter expression and further contribute to oncogenesis.

Our screening of OCSCC samples as well as matched and non-matched controls have identified distinct viral and other microbial signature patterns associated with OCSCC. We detect a distinct OCSCC microbiome signature consisting primarily of HPV16 viral signatures; bacterial signatures of Proteobacterias *Eshcherichia*, *Brevundimonas*, *Comamonas*, *Alcaligenes*, *Caulobacter*, *Cardiobacterium*, *Plesiomonas*, *Serratia*, *Edwardsiella*, *Haemophilus*, *Frateuria* along with Actinobacteria *Rothia* and Bacteroidetes *Peptoniphilus*; fungal signatures of *Rhodotorula*, *Geotrichum*, *Pneumocystis* and parasitic signatures of *Hymenolepis*, *Centrocestus*, *Trichinella* to be associated only with OCSCC and not the controls. This is an initial map of microbial association that can serve as potential diagnostic tools for OCSCC/OPSCCs. Importantly, we have also identified a microbial-host fusion map providing a more comprehensive map throughout the somatic human chromosomes. These integrations may alter host gene expression in ways that may promote OCSCC/OPSCC.

## Methods

### PathoChip design

The PathoChip Array design has been previously described^19, 20^. Briefly, the array was generated from a metagenome of 58 chromosomes in silico. It comprises of 60,000 probe sets of sequenced microorganisms in the Genbank, which are manufactured as SurePrint glass slide microarrays (Agilent Technologies Inc.), containing 8 replicate arrays per slide^20^. Each probe is a 60-nt DNA oligomer that targets multiple genomic regions of pathogenic viruses, prokaryotic, and eukaryotic microorganisms. The PathoChip technology, combined with PCR and NGS, is a valuable strategy for detecting and identifying pathogens in human cancers and other diseases^20^. Probes and accession annotations are available in the Gene Expression Omnibus (http://www.ncbi.nlm.nih.gov/geo/)^20^.

### Sample preparation and Microarray processing

PathoChip screening utilized both DNA and RNA extracted from formalin-fixed paraffin-embedded (FFPE) tumor tissues as described previously^20^. 100 de-identified FFPE oral cavity (OSCC) and oropharyngeal (OCSCC) squamous cell carcinoma samples, collectively referred here as OCSCC were received as 10 µm sections on non-charged glass slides and 20 each of matched and non-matched control samples were provided as paraffin rolls from the Abramson Cancer Center Tumor Tissue and Biosample Core. All the samples are de-identified tissues and thus there was no requirement for informed consent as it is deemed exempt according to Federal and University guidelines. Both the tumor and control tissues were read by a pathologist. Clinically normal samples adjacent to the cancers are referred here as “matched controls” as they were obtained from 20 cancer patients included in the study, while non-matched controls were oral tissues (uvula) obtained from otherwise healthy individuals. DNA and RNA were extracted in parallel^19, 20^ from rolls or mounted sections of each FFPE sample. The quality of extracted nucleic acids was determined by agarose gel electrophoresis and the A_260/280_ ratio. The extracted RNA and DNA samples were subjected to whole genome and transcriptome amplification (referred here as WTA) using TransPlex Complete Whole Transcriptome Amplification Kit (Sigma-Aldrich, St. Louis, MO) using 50 ng each of RNA and DNA as input and manufacturers protocol as described earlier^19, 20^. A total of 60 arrays were used to screen the 100 OCSCC/OPSCC samples, with 48 individual and the rest pooled in groups of 4–5 samples. The 20 matched and 20 non-matched control samples were pooled for screening using 4 arrays for each set of controls. The WTA products were analyzed by agarose gel electrophoresis and showed a range of 200–400 bp amplicon sizes. Human reference RNA and DNA were also extracted from the human B cell line, BJAB and 15 ng of each were used for WTA^19, 20^. The WTA products were purified, (PCR purification kit, Qiagen, Germantown, MD, USA), and 2 µg of the amplified products from the cancer tissues was labelled with Cy3 and that from the human reference was labelled with Cy5 (SureTag labeling kit, Agilent Technologies, Santa Clara, CA) as per manufacturer’s protocol^19, 20^. Human reference DNA and RNA was used to determine cross-hybridization of probes to human DNA. The labelled cDNA/DNAs were purified and the efficiencies of labeling were determined by measuring absorbance at 550 nm (for Cy3) and 650 nm (for Cy5). The labelled samples (Cy3 plus Cy5) were hybridized to the PathoChip as described previously^19, 20^. The hybridization cocktail (CGH blocking agent and hybridization buffer), was added to each of the labeled test sample (Cy3) mixed with reference (Cy5), denatured and hybridized to the arrays in 8-chamber gasket slides. The slides were incubated at 65 °C with rotation^19, 20^ and washed, then scanned for visualization using an Agilent SureScan G4900DA array scanner^19, 20^.

#### Microarray Data Extraction and Statistical analysis

The microarray data extraction and analyses have been described previously^19, 20^. The raw data from the microarray images were extracted using Agilent Feature Extraction software; normalization and data analyses were done in the Partek Genomics Suite (Partek Inc., St. Louis, MO, USA) as previously described^19, 20^. Model-based analysis of tiling arrays (MAT), which utilized a sliding window to scroll through the entire metagenome of the array to detect positive hybridization signal, was used to detect positive regions in the metagenome for each tumor^19, 20^. Analysis at the individual probe level (both for specific and conserved probes), and at the accession level (taking into account all the probes per accession), were performed as previously described^19, 20^. Probes of the microorganisms (microbial signatures) were detected in the samples by both outlier analyses (detecting probes in few samples) and paired t-tests with False Discovery Rate (FDR) multiple correction (detecting probes of significance in the majority of the tumor samples analyzed). The hybridization signal of each probe for each of the samples was given a score, and we summed the weighted score of each probe in all the 100 cancer samples, and also in 20 each of the controls. We ranked the microbial detections based on their hybridization signal (weighted score sum) and prevalence. We included in our study, the signatures detected at least in >20% of the samples screened. We also performed one sided t-tests to determine if cancer samples have significant detection of the candidate signature of organisms compared to the control (both matched and non-matched) samples. The cancer samples were also subjected to hierarchical clustering, based on the detection of microbial signatures in the samples, using the R program (Euclidean distance, complete linkage, non-adjusted values)^83, 84^, and the clusters were validated by CH index (Calinski and Harabasz index) which is implemented in R package as NbClust^85^. CH index is a cluster index that maximize inter-cluster distances and minimize intra-cluster distances. We calculated the possible cluster solution that would maximize the index values to achieve the best clustering of the data. The significant differences between the clusters observed by these methods were determined using t-test. Additional topological-based data analyses were conducted using the Ayasdi software (Ayasdi, Inc.), (Correlation metric, and L-infinity centrality lenses) where statistical significance between different groups was determined using two-sided t- test.

#### Probe Capture and Next Generation Sequencing

Probe Capture method has been previously described^19, 20^. Briefly, the WTA products of the oral cancer samples were pooled together for hybridization with selected biotinylated probes that were identified for microbial signatures in the oral cancer samples by the PathoChip screen. The targeted sequences were then captured by Streptavidin coated magnetic beads and libraries were generated for NGS. The selected probes were synthesized as 5′-biotinylated DNA oligomers (Integrated DNA Technologies, Coralville, IA, USA), mixed as 5 pools of capture probes (pools 1–5) (Fig. 5 and Table S3), and hybridized to WTA pools of oral cancer samples. Capture probe pool 1 contained 19 selected probes associated with bacteria (B capture), pool 2 contained 12 selected probes associated with the fungi (F capture), pool 3 contained 14 selected probes associated with parasitic signatures (P capture), pool 4 contains 36 other probes associated with viral and some bacterial signatures (O capture), pool 5 contains 6 HPV16 probes (HPV16 capture) (Table S3). Each of the 5 capture probe pools was added separately to the pooled WTA of the oral cancer samples (150ng/ul) in 5 separate reaction mixtures containing 3 M tetra-methyl ammonium chloride, 0.1% Sarkosyl, 50 mMTris-HCl, 4 mM EDTA, pH 8.0 (1XTMAC buffer). 5 target capture reactions were done (Table S3). The reaction mixtures were denatured (100 °C for 10 mins) followed by a hybridization step (60 °C for 3 hours). Streptavidin Dynabeads (Life Technologies, Carlsbad, CA, USA) were added with continuous mixing at room temperature for 2 hours, followed by three washes of the captured bead-probe-target complexes in 0.30 M NaCl plus 0.030 M sodium citrate buffer (2 × SSC) and three washes with 0.1 × SSC. Captured single-stranded target DNA was eluted in Tris-EDTA and used for library preparation using Nextera XT sample preparation kit (Illumina, San Diego, CA, USA)^19, 20^ followed by NGS. The 5 libraries were examined for quality control and submitted for NGS (Washington University Genome Technology Access Center, St. Louis, MO) using an Illumina MiSeq instrument with paired-end 250-nt reads. Adapters and low-quality fragments of raw reads were first removed using the Trim Galore software (http://www.bioinformatics.babraham.ac.uk/projects/trim_galore/). The processed reads were then aligned to the metagenome and the human genome using Genomic Short-read Nucleotide Alignment Program (GSNAP)^86, 87^ with default parameters. After alignment we employed featureCounts^88^ to count how many reads aligned to each of the capture probe regions. The detailed results for these capture probes are summarized in the table S4, and visualized in IGV^86^ (Figs 5 and S1a–f).

#### Microbial Fusion Detection

Prior to fusion detection, quality control of sequenced reads was applied. The Trim Galore software (http://www.bioinformatics.babraham.ac.uk/projects/trim_galore/) was employed for quality trimming of raw reads in order to remove adapters and low-quality fragments. We then used Virus-Clip^89^ to identify the virus fusion sites in the human genome. Specifically, we made use of the virus genome as the primary read alignment target, and first aligned reads to the PathoChip genome. Some mapped reads may contain soft-clipped segments. Soft-clipped reads were then extracted from the alignment and mapped (containing sequences of potential pathogen-integrated human loci) to the human genome. Utilizing this mapping information, the exact human and pathogen integration breakpoints at single-base resolution can be identified. All the integration sites were then automatically annotated with the affected human genes and their corresponding genomic co-ordinates.

Some of the host genes that supported viral/microbial genomic insertions by high sequence reads were subjected to Ingenuity Pathway Analysis (IPA) that helped to combine the host genes with knowledge extracted from the literatures to predict likely outcomes^90^. IPA software provided a statistical significance of the association of those genes with the disease outcome.

All the experiments were performed according to relevant guidelines and regulations as needed and according to all the licensing and approvals by institutional committees of Perelman School of Medicine at the University of Pennsylvania.

## Electronic supplementary material

Supplementary Information

Supplementary Table S5

Supplementary Table S6

Supplementary Table S7

Supplementary Table S8

Supplementary Table S9

Supplementary Table S10
