# Supplementary material for: Microbial Signatures Associated with Oropharyngeal and Oral Squamous Cell Carcinomas
Source: Sci Rep. 2017 Jun 22;7:4036. doi: 10.1038/s41598-017-03466-6 (PMC5481414; doi:10.1038/s41598-017-03466-6)
Supplement: Supplementary file 1 — Supplementary Information [file 41598_2017_3466_MOESM1_ESM.pdf]

Title: Microbial Signatures Associated with Oropharyngeal and Oral Squamous Cell Carcinomas

Sagarika Banerjee, Tian Tian, Zhi Wei, Kristen N Peck, Natalie Shih, Ara A Chalian, Bert W O'Malley Jr, Gregory S Weinstein, Michael D Feldman, James Alwine, Erle S. Robertson

Supplemental figure legends.

Figure S1. (Figure S1a-S1f). Probe capture sequencing alignments post MiSeq. The MiSeq reads from individual capture when aligned with the metagenome of PathoChip (Chip probes) was found to cluster mostly at the capture probe regions. The genomic location along with the number of MiSeq reads are mentioned in the figure. The alignment for O capture pool library is shown in Figure S1a; that of B library is shown Figure S1b & S1c; that of F library is shown in Figure S1d; that of P library is shown in Figure S1e & S1f.

Figure S1a

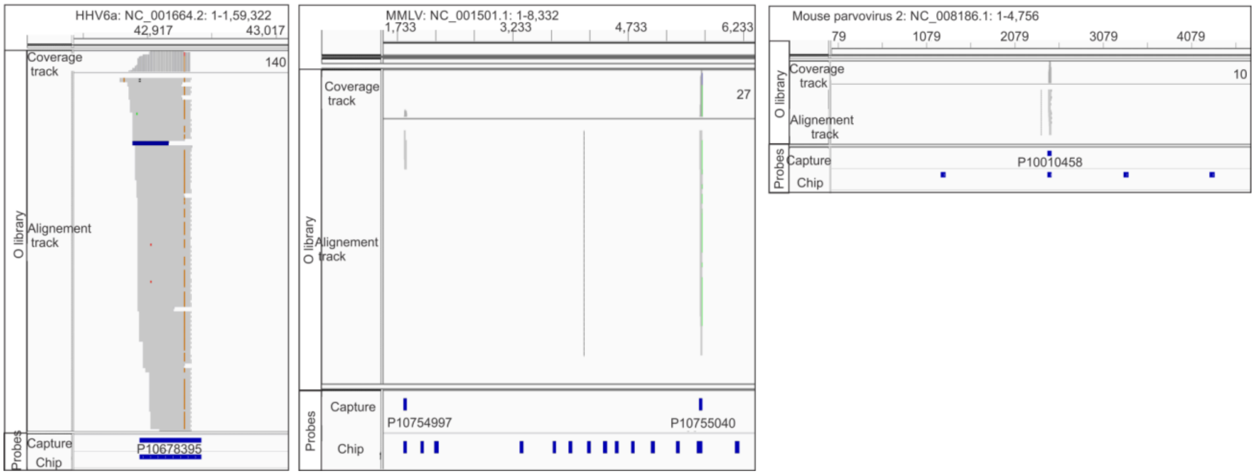

Figure S1b

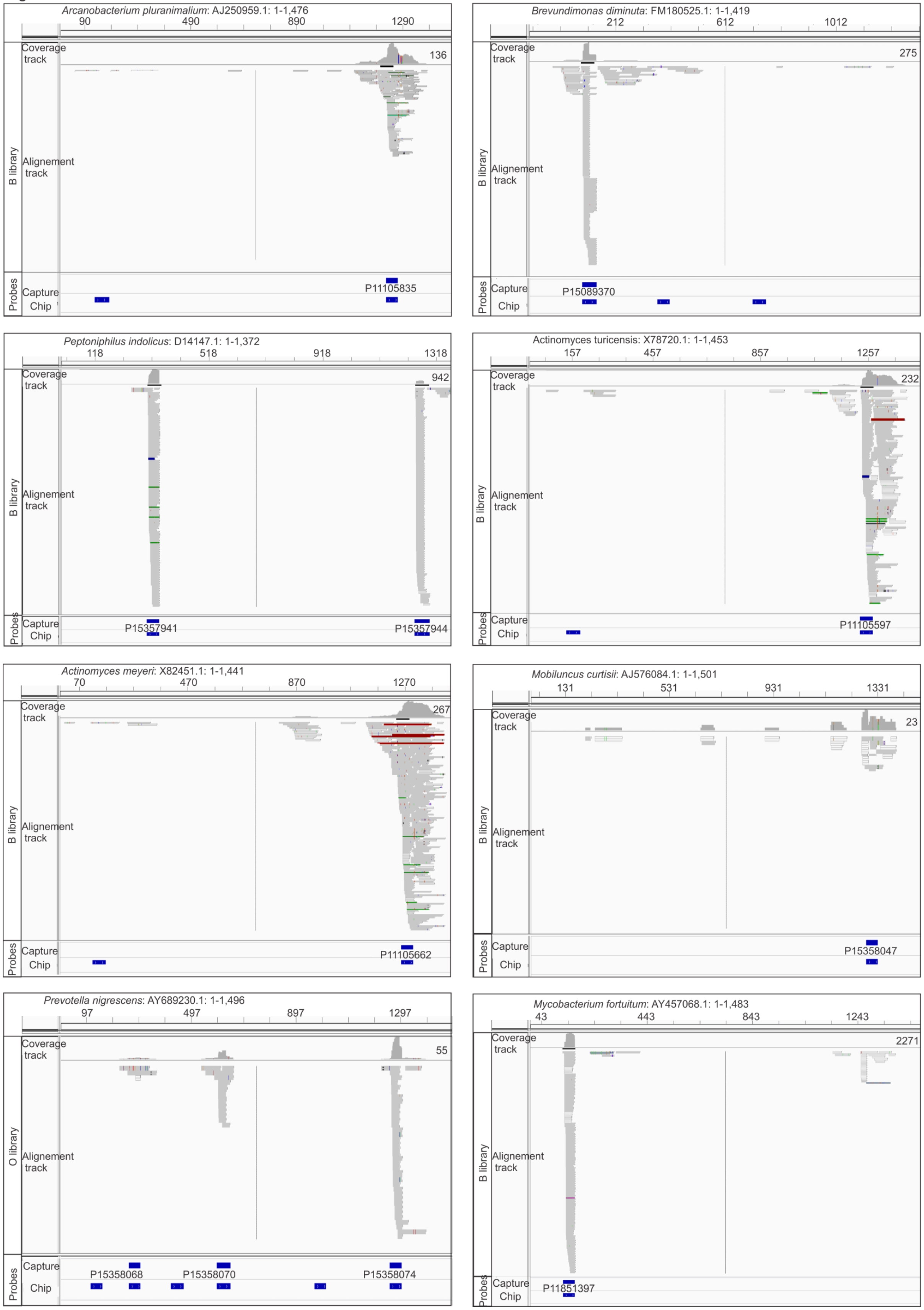

Arcanobacterium pluranimalium: AJ250959.1: 1-1,476
90
490
890
1290
Coverage track
136
Alignement track
B library
Probes
Capture
Chip
P11105835
Brevundimonas diminuta: FM180525.1: 1-1,419
212
612
1012
Coverage track
275
Alignement track
B library
Probes
Capture
Chip
P15089370
Peptoniphilus indolicus: D14147.1: 1-1,372
118
518
918
1318
Coverage track
942
Alignement track
B library
Probes
Capture
Chip
P15357941
P15357944
Actinomyces turicensis: X78720.1: 1-1,453
157
457
857
1257
Coverage track
232
Alignement track
B library
Probes
Capture
Chip
P11105597
Actinomyces meyeri: X82451.1: 1-1,441
70
470
870
1270
Coverage track
267
Alignement track
B library
Probes
Capture
Chip
P11105662
Mobiluncus curtisii: AJ576084.1: 1-1,501
131
531
931
1331
Coverage track
23
Alignement track
B library
Probes
Capture
Chip
P15358047
Prevotella nigrescens: AY689230.1: 1-1,496
97
497
897
1297
Coverage track
55
Alignement track
O library
Probes
Capture
Chip
P15358068
P15358070
P15358074
Mycobacterium fortuitum: AY457068.1: 1-1,483
43
443
843
1243
Coverage track
2271
Alignement track
B library
Probes
Capture
Chip
P11851397

Figure S1c

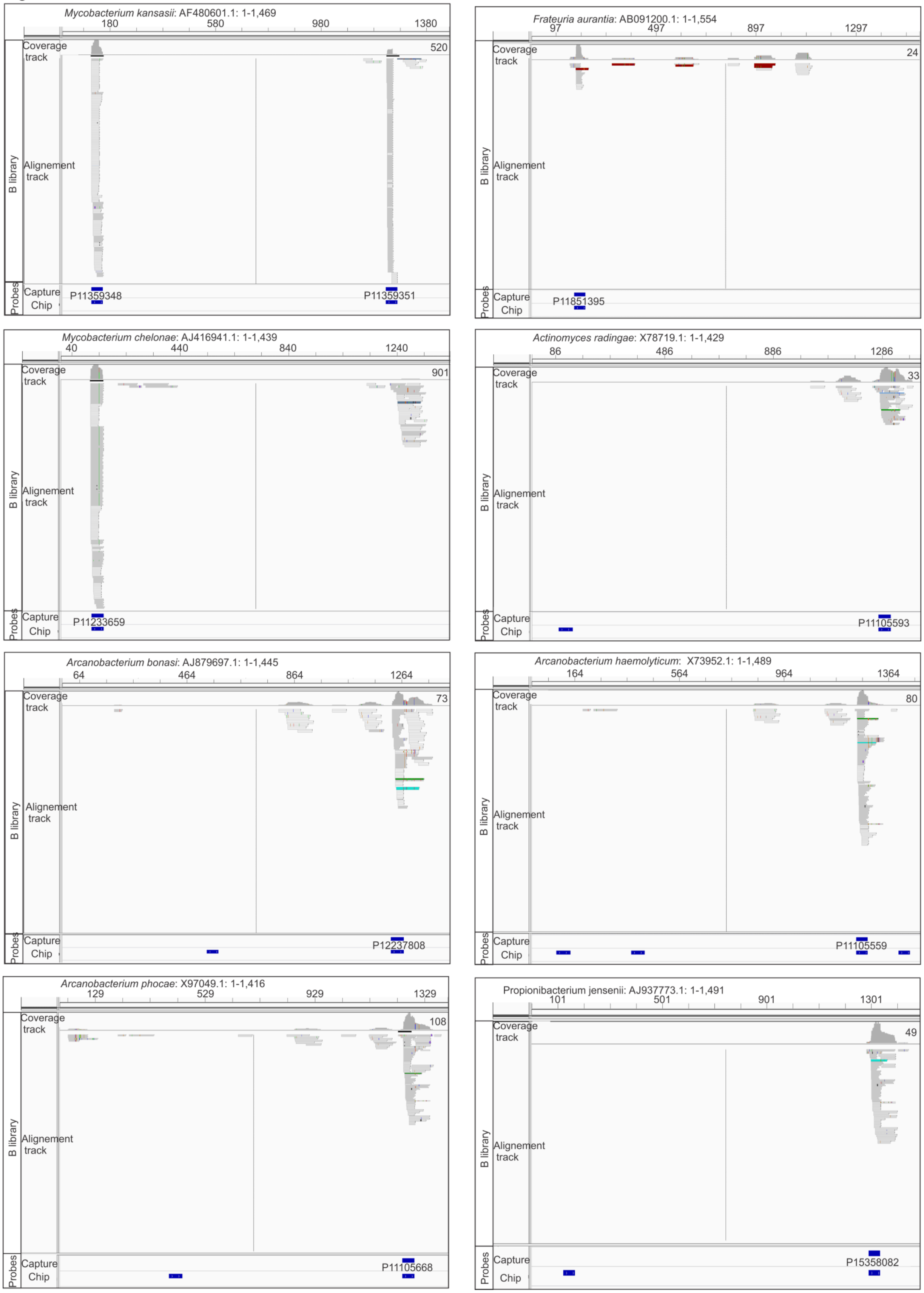

Mycobacterium kansasii: AF480601.1: 1-1,469
180
580
980
1380
Coverage track
520
B library
Alignement track
Probes
Capture Chip
P11359348
P11359351
Frateuria aurantia: AB091200.1: 1-1,554
97
497
897
1297
Coverage track
24
B library
Alignement track
Probes
Capture Chip
P11851395
Mycobacterium chelonae: AJ416941.1: 1-1,439
40
440
840
1240
Coverage track
901
B library
Alignement track
Probes
Capture Chip
P11233659
Actinomyces radingae: X78719.1: 1-1,429
86
486
886
1286
Coverage track
33
B library
Alignement track
Probes
Capture Chip
P11105593
Arcanobacterium bonasi: AJ879697.1: 1-1,445
64
464
864
1264
Coverage track
73
B library
Alignement track
Probes
Capture Chip
P12237808
Arcanobacterium haemolyticum: X73952.1: 1-1,489
164
564
964
1364
Coverage track
80
B library
Alignement track
Probes
Capture Chip
P11105559
Arcanobacterium phocae: X97049.1: 1-1,416
129
529
929
1329
Coverage track
108
B library
Alignement track
Probes
Capture Chip
P11105668
Propionibacterium jensenii: AJ937773.1: 1-1,491
101
501
901
1301
Coverage track
49
B library
Alignement track
Probes
Capture Chip
P15358082

Figure S1d

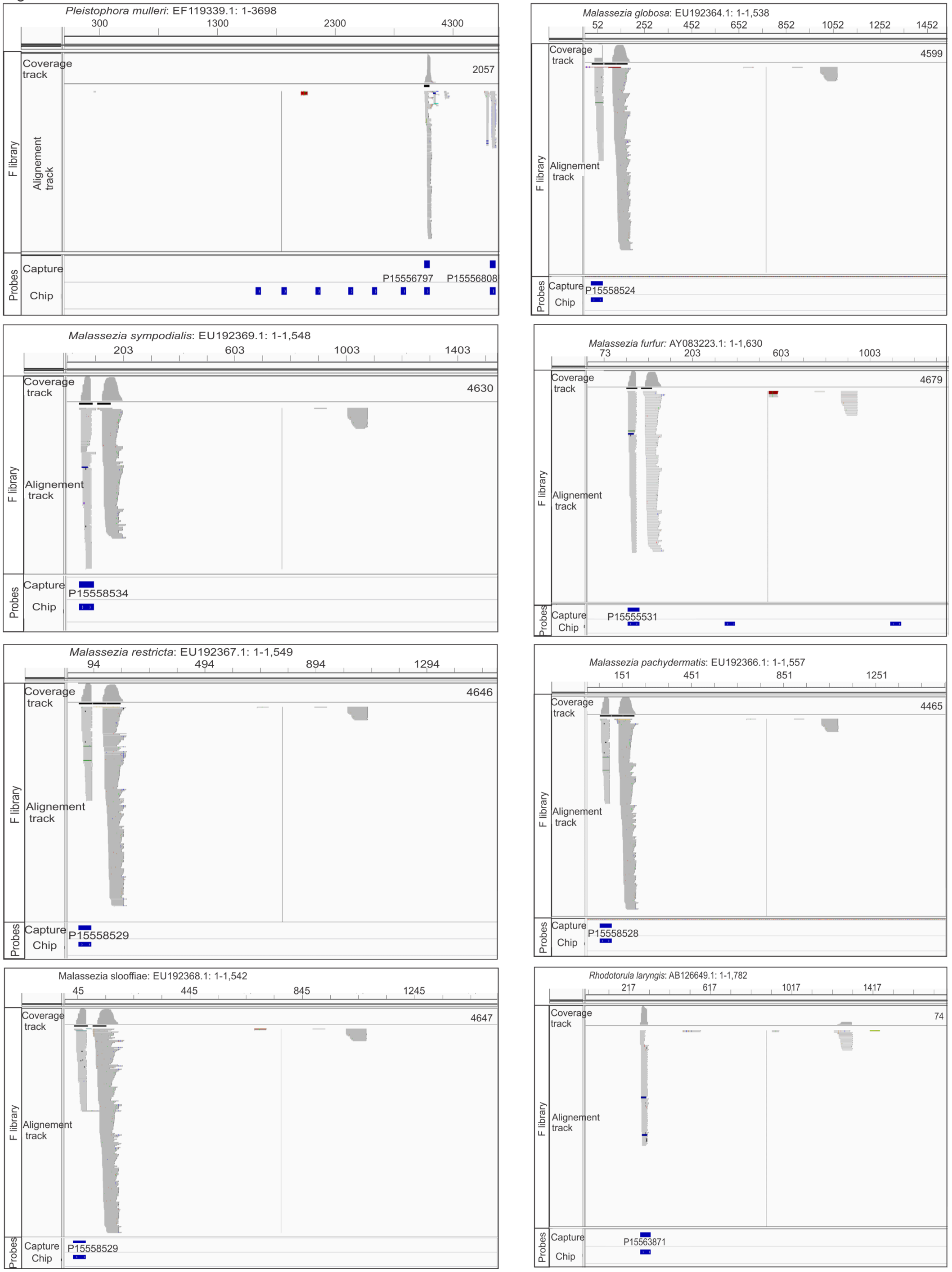

Pleistophora mulleri: EF119339.1: 1-3698
300
1300
2300
4300
Coverage track
2057
F library
Alignement track
Probes
Capture
Chip
P15556797
P15556808
Malassezia globosa: EU192364.1: 1-1,538
52
252
452
652
852
1052
1252
1452
Coverage track
4599
F library
Alignement track
Probes
Capture
Chip
P15558524
Malassezia sympodialis: EU192369.1: 1-1,548
203
603
1003
1403
Coverage track
4630
F library
Alignement track
Probes
Capture
Chip
P15558534
Malassezia furfur: AY083223.1: 1-1,630
73
203
603
1003
Coverage track
4679
F library
Alignement track
Probes
Capture
Chip
P15555531
Malassezia restricta: EU192367.1: 1-1,549
94
494
894
1294
Coverage track
4646
F library
Alignement track
Probes
Capture
Chip
P15558529
Malassezia pachydermatis: EU192366.1: 1-1,557
151
451
851
1251
Coverage track
4465
F library
Alignement track
Probes
Capture
Chip
P15558528
Malassezia slooffiae: EU192368.1: 1-1,542
45
445
845
1245
Coverage track
4647
F library
Alignement track
Probes
Capture
Chip
P15558529
Rhodotorula laryngis: AB126649.1: 1-1,782
217
617
1017
1417
Coverage track
74
F library
Alignement track
Probes
Capture
Chip
P15563871

Figure S1e

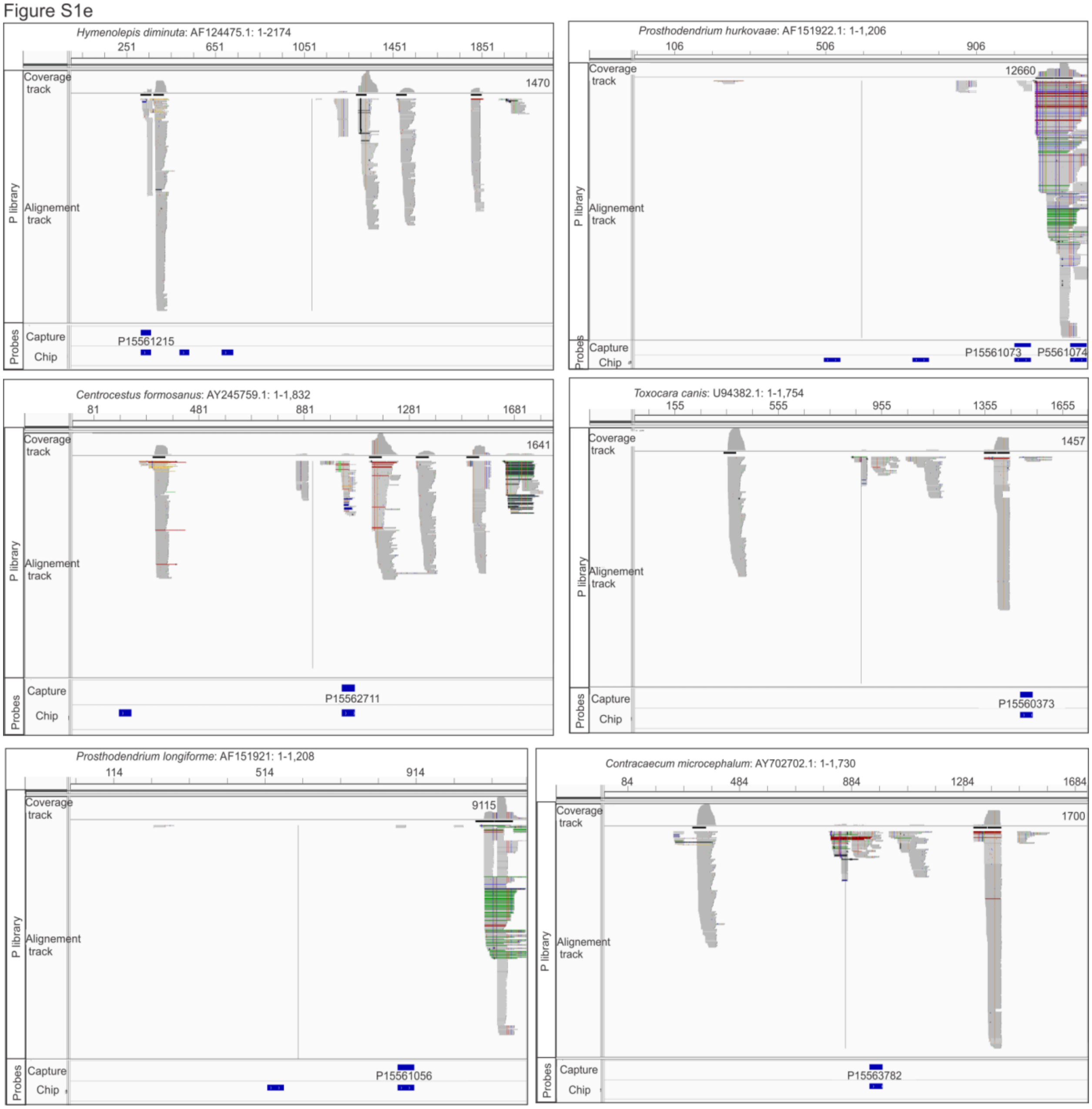

Hymenolepis diminuta: AF124475.1: 1-2174
251
651
1051
1451
1851
Coverage track
1470
P library
Alignement track
Probes
Capture
P15561215
Chip
Prosthodendrium hurkovaae: AF151922.1: 1-1,206
106
506
906
Coverage track
12660
P library
Alignement track
Probes
Capture
P15561073
P5561074
Chip
Centrocestus formosanus: AY245759.1: 1-1,832
81
481
881
1281
1681
Coverage track
1641
P library
Alignement track
Probes
Capture
P15562711
Chip
Toxocara canis: U94382.1: 1-1,754
155
555
955
1355
1655
Coverage track
1457
P library
Alignement track
Probes
Capture
P15560373
Chip
Prosthodendrium longiforme: AF151921: 1-1,208
114
514
914
Coverage track
9115
P library
Alignement track
Probes
Capture
P15561056
Chip
Contracaecum microcephalum: AY702702.1: 1-1,730
84
484
884
1284
1684
Coverage track
1700
P library
Alignement track
Probes
Capture
P15563782
Chip

Figure S1f

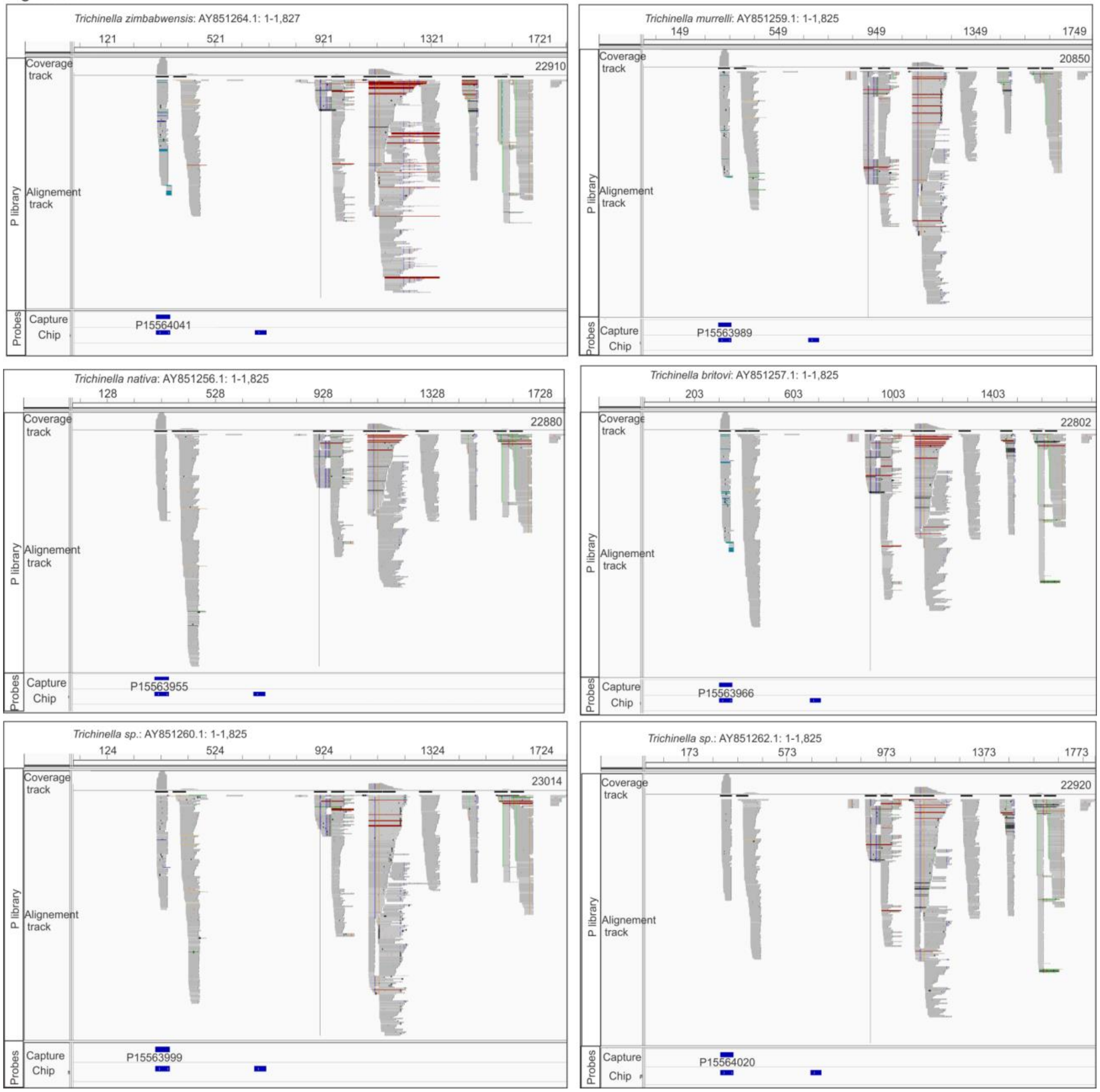
Trichinella zimbabwensis: AY851264.1: 1-1,827
121
521
921
1321
1721
Coverage track
22910
P library
Alignement track
Probes
Capture
Chip
P15564041
Trichinella murrelli: AY851259.1: 1-1,825
149
549
949
1349
1749
Coverage track
20850
P library
Alignement track
Probes
Capture
Chip
P15563989
Trichinella nativa: AY851256.1: 1-1,825
128
528
928
1328
1728
Coverage track
22880
P library
Alignement track
Probes
Capture
Chip
P15563955
Trichinella britovi: AY851257.1: 1-1,825
203
603
1003
1403
Coverage track
22802
P library
Alignement track
Probes
Capture
Chip
P15563966
Trichinella sp.: AY851260.1: 1-1,825
124
524
924
1324
1724
Coverage track
23014
P library
Alignement track
Probes
Capture
Chip
P15563999
Trichinella sp.: AY851262.1: 1-1,825
173
573
973
1373
1773
Coverage track
22920
P library
Alignement track
Probes
Capture
Chip
P15564020

Figure S2. Schematic representation of viral, bacterial, fungal and parasitic genomic insertional sites in human chromosomes. The genomic co-ordinates of the pathogens integrated and that of the host chromosome integration sites are mentioned. The co-ordinates for human chromosomes are from GRCh37/hf19 Assembly.

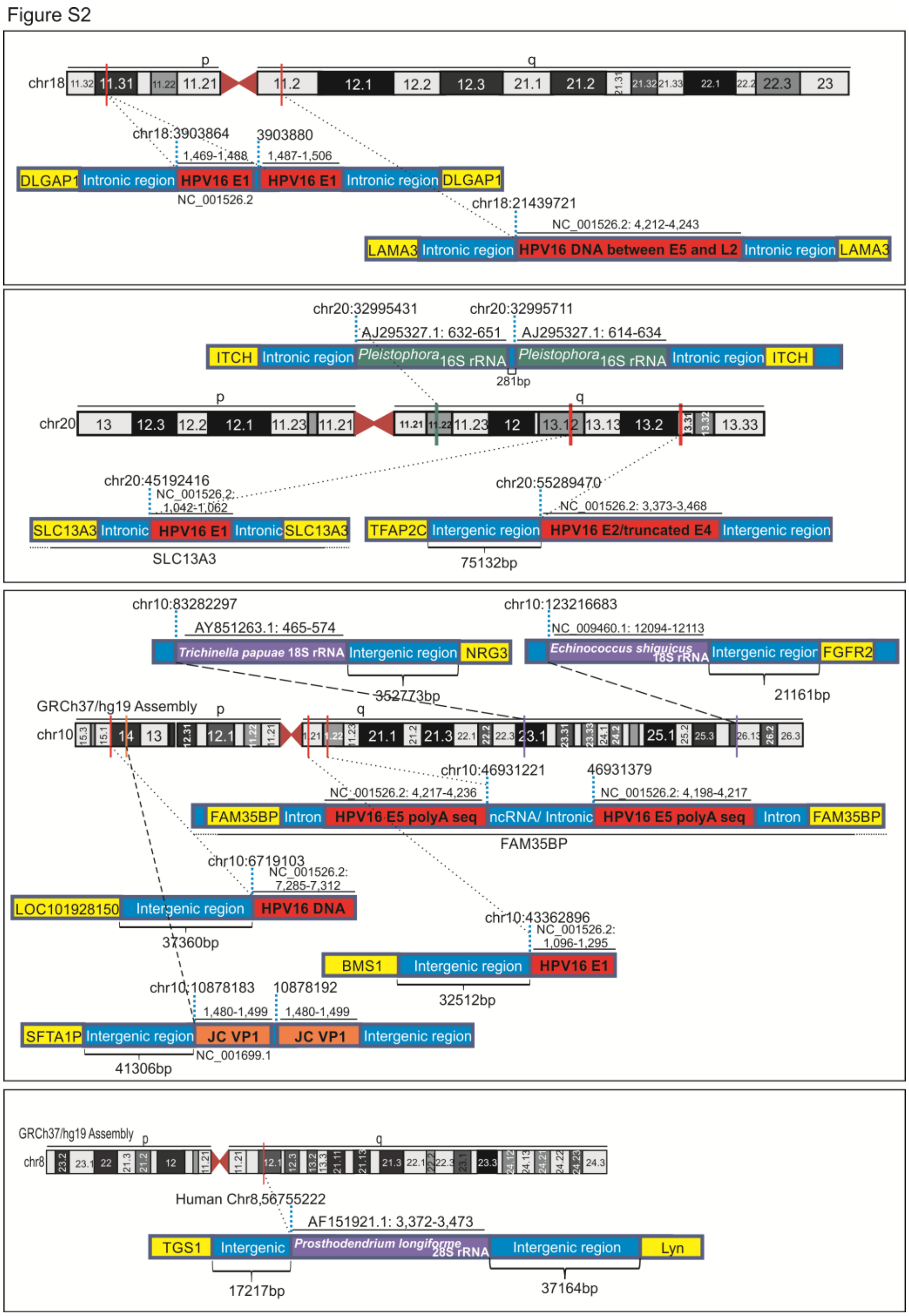

Title: Microbial Signatures Associated with Oropharyngeal and Oral Squamous Cell Carcinomas

Sagarika Banerjee, Tian Tian, Zhi Wei, Kristen N Peck, Natalie Shih, Ara A Chalian, Bert W O'Malley Jr, Gregory S Weinstein, Michael D Feldman, James Alwine, Erle S. Robertson

Supplemental table legends.

Table S1. Tumor location and HPV16 positivity status of the OSCC samples screened by PathoChip.

Table S2. List of the capture probe sequences used for capture sequencing.

Table S3. Microbial signatures detected in OSCC and control samples.

Table S4. MiSeq reads aligning to capture probe locations. The total number of MiSeq reads per capture pool for each accessions are mentioned. For example Bcounts for each accession designates the no. of reads in B library that aligned to the particular accession, of which Bcounts.probe designates the no. of reads that aligned to the capture probe location of that particular accession and Bcounts.not.probe designates the no. of reads aligning outside the capture probe region for that particular accession. Reads are mentioned similarly for F, P, O and HPV16 library as well.

Table S5. Statistical significance between cluster 1 and cluster 2 oral cancer samples that are clustered by NBClust software. The significant differences between the clusters observed by these methods were determined using two-sided t-test.

Table S6. Statistical significance between 'group a', 'group b' and 'un-ungrouped' oral cancer samples that are obtained by topological-based data analyses using Ayasdi software. The significant differences between the clusters observed were determined using two-sided t-test.

Table S7-S10. Gene fusion results. List of viral (Table S7), bacterial (Table S8), fungal (Table S9) and parasitic (Table S10) genomic regions integrated either in intronic, exonic, downstram or upstream of certain human genomic regions. Point of integration with genomic co-ordinates, both for the pathogen and human genome are mentioned. The number of reads for such kind of fusion are also mentioned. The description of the gene fusion results are as follows: Column A-H:type of left/right element of fusion fragment (micro-organism or human), left/right chromosome name (pathochip genome or human), left/right start and end co-ordinate. Column I: number of reads supporting fusion; Column J: Gene_region, indicating region (like intergenic, intronic, exon etc) of the fusion site in human genome; Column K: affected human gene name; Column L: Microbial genomic insertion position in human chromosome; Column M: Number of read support for human sequences; Column N: (Virus/bacteria/parasite/fungus).gene start coordinate of fusion organism in pathochip metagenome; Column O: (Virus/bacteria/parasite/fungus)_ coordinate of fusion site in pathochip metagenome; Column P: integration site of (Virus/bacteria/parasite/fungus)_coordinates at their genome; Column Q: (Virus/bacteria/parasite/fungus) region integrated; Column E: Accession number of microorganism integrated (description of accession).

Table S1

Study Code	Tumor location	Tumor type	p16 status
ENT01	tongue/tonsil	Sqaumous Cell Carcinoma (SCC)	Positive
ENT02	tongue/tonsil	SCC	Positive
ENT03	tonsil	SCC	Positive
ENT04	tonsil/base of tongue	SCC	Positive
ENT05	tonsil	SCC	Positive
ENT06	tonsil	SCC with sarcomatoid growth	Positive
ENT07	tonsil	SCC	Positive
ENT08	tonsil	SCC	Positive
ENT09	tonsil/tongue base	SCC	Positive
ENT10	floor of mouth/cheek	SCC	Negative
ENT11	tonsil/tongue base	SCC	Positive
ENT12	tonsil/tongue base	SCC	Positive
ENT13	tonsil	SCC	Positive
ENT14	oropharynx/palatine tonsil	SCC	Positive
ENT15	base of tongue	SCC	Negative
ENT16	tongue base	SCC	Positive
ENT17	tonsil/pharynx	SCC	Positive
ENT18	tonsil	SCC	Positive
ENT19	tonsil	SCC	Positive
ENT20	tonsil	SCC	Positive
ENT21	tongue base	SCC	Positive
ENT22	tonsil	SCC	Positive
ENT23	tonsil	SCC	Positive
ENT24	tongue base	SCC	Positive
ENT25	tongue base	SCC	Negative
ENT26	tonsil	SCC	Positive
ENT27	palatine tonsil	SCC	Positive
ENT28	oropharynx/base of tongue including lingual tonsil	SCC	Positive
ENT29	oropharynx	SCC	Positive
ENT30	Retromolar trigone	SCC with clear cell change	Positive
ENT31	oropharynx/palatine tonsil	SCC	Positive
ENT32	tongue/floor of mouth	SCC	Negative
ENT33	Tonsil	SCC	Positive
ENT34	tonsil/tongue base	SCC	Positive
ENT35	tonsil	Poorly differentiated carcinoma	Positive
ENT36	retromolar trigone/palate	SCC	Positive
ENT37	pharynx	SCC	Negative
ENT38	buccal mucosa	SCC	Negative
ENT39	tonsil	SCC	Positive
ENT40	tonsil	SCC	Positive
ENT41	base of tongue inclusing lingual tonsil	SCC	Positive
ENT42	tonsil	SCC	Positive
ENT43	tonsil	SCC	Positive
ENT44	oropharynx, base of tongue including lingual tonsil	SCC	Positive
ENT45	oropharynx, palatine tonsil	SCC	Positive
ENT46	base of tongue, including lingual tonsil	SCC	Positive
ENT47	base of tongue including lingual tonsil	SCC	Negative
ENT48	tongue	SCC	Negative
ENT49	oropharynx/palatine tonsil, base of tongue including lingual tonsil	SCC	Positive
ENT50	palatine tonsil, base of tongue including lingual tonsil	SCC	Positive
ENT51	oropharynx/palatine tonsil, base of tongue including lingual tonsil	SCC	Positive
ENT52	tonsillar pillar	SCC	Negative
ENT53	oropharynx/palatine tonsil, base of tongue including lingual tonsil	SCC	Positive
ENT54	tongue/floor of mouth	SCC	Negative
ENT55	oropharynx/palatine tonsil	SCC	Positive
ENT56	oropharynx/soft palate	SCC	Positive
ENT57	tonsil	SCC	Positive
ENT58	oropharynx/base of tongue including lingual tonsil	SCC	Positive
ENT59	oropharynx/base of tongue including lingual tonsil	SCC	Positive
ENT60	oropharynx/palatine tonsil, base of tongue including lingual tonsil/pharyngeal wall (posterior)	SCC	Positive
ENT61	tonsil	SCC	Positive
ENT62	base of tongue inclusing lingual tonsil	SCC	Positive
ENT63	oropharynx base of tongue, including lingual tonsil	SCC	Positive
ENT64	oropharynx base of tongue, including lingual tonsil	SCC	Positive
ENT65	oropharynx base of tongue, including lingual tonsil	SCC	Positive
ENT66	palatine tonsil	SCC	Positive
ENT67	oropharynx palatine tonsil base of tongue, including lingual tonsil	SCC	Positive
ENT68	tonsil, base of tongue	SCC	Positive
ENT69	hard palate	SCC	Positive
ENT70	tonsil	SCC	Positive
ENT71	tonsil	SCC	Positive
ENT72	tongue base	SCC	Positive
ENT73	tonsil, tongue base	SCC	Negative
ENT74	tonsil and tongue base	SCC	Positive
ENT75	tonsil	SCC	Positive
ENT76	oropharynx base of tongue, including lingual tonsil, pharyngeal wall (posterior)	SCC	Positive
ENT77	tonsil ,tongue base, pharynx	SCC	Positive
ENT78	oropharynx palatine tonsil	SCC	Positive
ENT79	tonsil/tongue base	SCC	NA
ENT80	tongue base	SCC	NA
ENT81	tonsil/base of tongue	SCC	NA
ENT82	tongue base	SCC	Positive
ENT83	tonsil	SCC	Positive
ENT84	buccal mucosa	SCC	NA
ENT85	tongue	SCC	Negative
ENT86	oropharynx, tonsil	SCC	Positive
ENT87	oropharyx, tongue base	SCC	Positive
ENT88	tonsil/tongue base	SCC	Positive
ENT89	tonsil	SCC	Positive
ENT90	tonsillar pillar	SCC	Positive
ENT91	tongue base	SCC	Positive
ENT92	tongue base	SCC	Positive
ENT93	tonsil	SCC	Positive
ENT94	tonsil	SCC	NA
ENT95	left cheek and lip resection	SCC	NA
ENT96	Left radical tonsillectomy, tongue base resection	SCC	Positive
ENT97	Tongue, right	SCC	NA
ENT98	Left radical tonsillectomy, tongue base resection, limited pharyngectomy	SCC	Positive
ENT99	Left tonsil, left radical tonsillectomy, tongue base resection, limited pharyngectomy	SCC	Positive
ENT100	Right tonsillectomy, tongue base resection	SCC	NA

Table S2. Microbial signatures detected in OSCC and control samples.

	Cancer	MC	NC	Cancer/MC	Cancer/NC	Cancer/MC/NC	NC/MC
Viral signatures	**HPV16**	Coronaviridae	Togaviridae			Papillomaviridae	Adenoviridae
						Poxviridae	Picornaviridae
						Retroviridae	Flaviviridae
						Polyomaviridae	Hepadnaviridae
						Herpesviridae	Parvoviridae
						Reoviridae	Anelloviridae
						Orthomyxoviridae	
Bacterial signatures	***Eshcherichia***	*Campylobacter*	*Porphyromonas*	***Arcanobaterium***	*Mobiluncus*	*Mycobacterium*	
	Rothia	*Streptobacillus*		***Actinomyces***	*Propionibacterium*	*Sphingobacterium*	
	Peptoniphilus			***Aeromonas***	*Prevotella*	*Streptococcus*	
	Brevundimonas			***Bordetella***	*Citrobacter*		
	Comamonas			***Aerococcus***	*Sphingomonas*		
	Alcaligenes			***Pediococcus***	*Peptostreptococcus*		
	Caulobacter			***Acinetobacter***			
	Cardiobacterium			***Actinobacillus***			
	Plesiomonas			***Veillonella***			
	Serratia						
	Edwardsiella						
	Haemophilus						
	Frateuria						
Fungal signatures	***Rhodotorula***	*Pseudallescheria*	*Piedraia*	***Fonsecaea***		*Malassezia*	*Microsporum*
	Geotrichum		*Trichosporon*			*Pleistophora*	*Alternaria*
	Pneumocystis		*Encephalitozoon*			*Absidia*	
			Trichophyton			*Phialophora*	
			Paecilomyces			*Cladophialophora*	
			Madurella			*Cladosporium*	
Parasitic signtures	***Hymenolepis***	*Echinococcus*	*Dipylidium*		*Prosthodendrium*	*Toxocara*	*Diphyllobothrium*
	Centrocestus		*Anisakis*		*Contracaecum*		*Strongyloides*
	Trichinella		*Echinostoma*				

Table S3

<table>
<tr><th>Capture Probe Library</th><th>Microorganisms</th><th>Probe sequences used for capture 5'-3'</th></tr>
<tr><td rowspan="19">B (Bacterial pool)</td><td>*|AJ250959.1|Arcanobacterium sp.*</td><td>/5Biosg/GAATCCCTTAAAGCCAGTCTCAGTTCGGATTGGGGTCTGCAACTC</td></tr>
<tr><td>*|X73952.1|Arcanobacterium haemolyticum*</td><td>/5Biosg/GAATCCCTTAAAGCCAGTCTCAGTTCGGATTGGGGTCTGCAACTC</td></tr>
<tr><td>*|X97049.1| Arcanobacterium phocae*</td><td>/5Biosg/GAATCCCTTAAAGCCAGTCTCAGTTCGGATTGGGGTCTGCAACTC</td></tr>
<tr><td>*|AJ879697.1| Arcanobacterium sp.*</td><td>/5Biosg/CTAATCACTTAAAGTCTGTCTCAGTTCGGATTGGGGTCTGCAACTCGAC</td></tr>
<tr><td>*|AJ576084.1| Mobiluncus curtisii*</td><td>/5Biosg/CAACTCGACCCCATGAAGTTGGAGTCGCTAGTAATCGCAGATCAG</td></tr>
<tr><td>*|J01859.1| Esherichia coli*</td><td>/5Biosg/TAAAACTCAAATGAATTGACGGGGGCCCGCACAAGCGGTGGAGCATGTGGTTTAATTCGA</td></tr>
<tr><td>*|X78719.1|Actinomyces radingae*</td><td>/5Biosg/ATCGGGGTCTGCAACTCGACCTCGTGAAGGTGGAGTCGCTAGTAAT</td></tr>
<tr><td>*|X78720.1|Actinomyces turicensis*</td><td>/5Biosg/ATATCGTAAGGTGGAGCGAATCACTTAAAGCCAGTCTCAGTTCGGATTG</td></tr>
<tr><td>*|X82451.1|Actinomyces meyeri*</td><td>/5Biosg/GAATCCCTTAAAGCCAGTCTCAGTTCGGATTGGGGTCTGCAACTC</td></tr>
<tr><td>*|AJ717364.1| Rothia dentocariosa*</td><td>/5Biosg/CTAATCCCTAAAAGCCGGTCTCAGTTCGGATTGGGGTCTGCAACT</td></tr>
<tr><td>*|AJ937773.1|Propionibacterium jensenii*</td><td>/5Biosg/GAATCTCAAAAAGCCAGTCTCAGTTCGGATTGGGGTCTGCAACTC</td></tr>
<tr><td>*|D14147.1|Peptoniphilus indolicus*</td><td>/5Biosg/CCTGAGAGGGTGAACGGCCACATTGGAACTGAGAAACGGTCCAAA</td></tr>
<tr><td>*|D14147.1|Peptoniphilus indolicus*</td><td>/5Biosg/GATAGCAAGCGAATCTCAAAAAGCCTATCTCAGTTCGGATTGTTCTCTGCAACT</td></tr>
<tr><td>*|AF480601.1| Mycobacterium kansasii*</td><td>/5Biosg/CGGGATAAGCCTGGGAAACTGGGTCTAATACCGGATAGGACCACT</td></tr>
<tr><td>*|AF480601.1| Mycobacterium kansasii*</td><td>/5Biosg/CGAGGTTAAGCGAATCCTTTTAAAGCCGGTCTCAGTTCGGATCGG</td></tr>
<tr><td>*|AJ416941.1| Mycobacterium chelonae*</td><td>/5Biosg/ATAAGCCTGGGAAACTGGGTCTAATACCGGATAGGACCACACACTT</td></tr>
<tr><td>*|AY457068.1| Mycobacterium fortuitum*</td><td>/5Biosg/CACTTTGGGATAAGCCTGGGAAACTGGGTCTAATACCGAATAGGAC</td></tr>
<tr><td>*|FM180525.1| Brevundimonas diminuta*</td><td>/5Biosg/GAGTTGCAGAGGACAATCCGAACTGAGACAATTTTAAGGATTAACCCTCTGTAG</td></tr>
<tr><td>*|AB091200.1|Frateuria aurantia*</td><td>/5Biosg/GAAACTTACGCTAATACCGCATACGTCCTACGGGAGAAAGCAGGG</td></tr>
<tr><td rowspan="12">F (Fungal pool)</td><td>*|AB126649.1| Rhodotorula laryngis*</td><td>/5Biosg/CTTCATTCAAATACCTGCCCTATCAACTTTCGATGGTAGGATAGAGGCCTAC</td></tr>
<tr><td>*|DQ325447.1|Geotrichum citri-aurantii*</td><td>/5Biosg/AGAGCTCTTTCATGATTTTGTGGGTGGTGGTGCATGGCCGTTCTTAGTTGGTGGAGTGAT</td></tr>
<tr><td>*|EU192364.1|Malassezia globosa*</td><td>/5Biosg/GTGCTGGCGATACTTCATTCAAATATCTGCCCTATCAACTGTCGATGGTAG</td></tr>
<tr><td>*|AY083223.1| Malassezia furfur*</td><td>/5Biosg/TTCATTCAAATATCTGCCCTATCAACTGTCGATGGTAGGATAGAGGCCTACCAT</td></tr>
<tr><td>*|EU192366.1|Malassezia pachydermatis*</td><td>/5Biosg/ATGCTTCATTCAAATATCTGCCCTATCAACTGTCGATGGTAGGATAGAGGCCTA</td></tr>
<tr><td>*|EU192367.1|Malassezia restricta*</td><td>/5Biosg/CTGGCGGTGCTTCATTCAAATATCTGCCCTATCAACTGTCGATGGTAG</td></tr>
<tr><td>*|EU192368.1|Malassezia slooffiae*</td><td>/5Biosg/CGGGGGTGCTTCATTCAAATATCTGCCCTATCAACTGTCGATGGTAG</td></tr>
<tr><td>*|EU192369.1|Malassezia sympodialis*</td><td>/5Biosg/ATGCTTCATTCAAATATCTGCCCTATCAACTGTCGATGGTAGGATAGAGGCCTA</td></tr>
<tr><td>*|EF119339.1|Pleistophora mulleri*</td><td>/5Biosg/GAGGAAATAACTGTAAGCACGGGTTAACGGCGGGAGTAACTATGACTC</td></tr>
<tr><td>*|EF119339.1|Pleistophora mulleri*</td><td>/5Biosg/GCAGAAAGCGACGGTGCTTTTTGATTCTTCGATTCGACTCTTCCTATCATG</td></tr>
<tr><td>*|AF044392.1|Pleistophora ehrenbaumi*</td><td>/5Biosg/AAAGCACAGGAAGGAAGGGTCAAGAACAGTCAGTGATGCCCTTAG</td></tr>
<tr><td>*|AB266392.1|Pneumocystis jirovecii*</td><td>/5Biosg/CTTGTGCTGGCGATGGTTCATTCAAATTTCTGCCCTATCAACTTTCGATG</td></tr>
<tr><td rowspan="14">P (Parasitic pool)</td><td>*|AF124475.1| Hymenolepis diminuta*</td><td>/5Biosg/CTTCAAATGTCTGCCCTATCAACTTTCGATGGTAGGTCACCTGCCTAC</td></tr>
<tr><td>*|AY245759.1|Centrocestus formosanus*</td><td>/5Biosg/AAAGTCAGAGGTTCGAAGACGATCAGATCCGTCCTAGTTCTGACCATAAC</td></tr>
<tr><td>*|AF151921.1|Prosthodendrium longiforme*</td><td>/5Biosg/GTCTTGAAACACGGACCAAGGAGAGTAACATGCGCGCGAGTCATT</td></tr>
<tr><td>*|AF151922.1|Prosthodendrium hurkovaae*</td><td>/5Biosg/GAGCGGTAGGCGCATCACCGGCCCGTCCCATGACAATTGTTCTTG</td></tr>
<tr><td>*|AF151922.1|Prosthodendrium hurkovaae*</td><td>/5Biosg/CCGAAGCGGTTCTGACGTGCAAATCGATCGCCAGACATGAGTATA</td></tr>
<tr><td>*|AY851256.1| Trichinella nativa*</td><td>/5Biosg/ACATCACTTTCAAGTGTCTGCCTTATCAACCTTCGATGGTAGTCTATGCGACTA</td></tr>
<tr><td>*|AY851257.1| Trichinella britovi*</td><td>/5Biosg/ACATCACTTTCAAGTGTCTGCCTTATCAACCTTCGATGGTAGTCTATGCGACTA</td></tr>
<tr><td>*|AY851259.1|Trichinella murrelli*</td><td>/5Biosg/ACATCACTTTCAAGTGTCTGCCTTATCAACCTTCGATGGTAGTCTATGCGACTA</td></tr>
<tr><td>*|AY851260.1| Trichinella sp.*</td><td>/5Biosg/ACATCACTTTCAAGTGTCTGCCTTATCAACCTTCGATGGTAGTCTATGCGACTA</td></tr>
<tr><td>*|AY851262.1|Trichinella sp.*</td><td>/5Biosg/ACATCACTTTCAAGTGTCTGCCTTATCAACCTTCGATGGTAGTCTATGCGACTA</td></tr>
<tr><td>*|AY851263.1|Trichinella papuae*</td><td>/5Biosg/ACATCACTTTCAAGTGTCTGCCTTATCAACCTTCGATGGTAGTCTATGCGACTA</td></tr>
<tr><td>*|AY851264.1|Trichinella zimbabwensis*</td><td>/5Biosg/ACATCACTTTCAAGTGTCTGCCTTATCAACCTTCGATGGTAGTCTATGCGACTA</td></tr>
<tr><td>*|AY702702.1|Contracaecum microcephalum*</td><td>/5Biosg/GATCAGATACCGCCCTAGTTCTGACCGTAAACGATACCAACTAGCGTT</td></tr>
<tr><td>*|U94382.1|Toxocara canis*</td><td>/5Biosg/TAACCCGTTGAAAATCCTCCGTGATCGGGATCGGGAATTGCAATTATTT</td></tr>
<tr><td rowspan="27">O (Other pool)</td><td>|NC_008186.1| Mouse parvovirus 2</td><td>/5Biosg/AAAGACGCTAAAGACTGGGGAGGCAAGGTTGGTCACTACTTCTTTAGAA</td></tr>
<tr><td>|NC_001664.2| Human herpesvirus 6A</td><td>/5Biosg/TATAGCAGCGCCCGATGCCAACGCGACAGGGTGAGTCACATAGAT</td></tr>
<tr><td>|NC_001664.2| Human herpesvirus 6A</td><td>/5Biosg/CAATACAGAGGTTGTGTTTGGCAAAATTTAAGCAGCAATTGTTGTATCTAAATATTAAGG</td></tr>
<tr><td>|NC_006879.1| Simian adenovirus 1</td><td>/5Biosg/TCTTGCATCAGTCTCTCCACCGGAACCTCTGCTTCTCCCCTGTCT</td></tr>
<tr><td>|NC_006879.1| Simian adenovirus 1</td><td>/5Biosg/AGAGGCCAGAGAGGAGCCAACTGGCAAAGCACGCTGAACAGCATC</td></tr>
<tr><td>|NC_002657.1| Classical swine fever virus</td><td>/5Biosg/ATTGGGGAACAAGCCCAGAGAAGGGGGAGAGTTGGGAGAGTAAAG</td></tr>
<tr><td>|NC_001699.1| JC polyomavirus</td><td>/5Biosg/TAACAGCCAGTAAACAAAGCACAAGGGGAAGTGGAAAGCAGCCAA</td></tr>
<tr><td>|NC_001699.1| JC polyomavirus</td><td>/5Biosg/AAAGGGAGGGAACCTATATTTCTTTTGGCCACTCATACACCCAAAGTATAGATGATG</td></tr>
<tr><td>|NC_001699.1| JC polyomavirus</td><td>/5Biosg/TTATAGGGGTGACAAGTTTGATGAATGTGCACTCTAATGGGCAAGCAACTCAT</td></tr>
<tr><td>|NC_001664.2| Human herpesvirus 6A</td><td>/5Biosg/CTAAAACCGTCTTTGCCTCTCAAATCTTTAATGAGAAAAACGTTACGGGGTGAAAC</td></tr>
<tr><td>|NC_001664.2| Human herpesvirus 6A</td><td>/5Biosg/ACATTAATACAGGCAAAGGACACAATTCACGGCATTCTTGAAGCCGTTGAC</td></tr>
<tr><td>|NC_001664.2| Human herpesvirus 6A</td><td>/5Biosg/CTATCAGAATTACGAACGCCCCGATAAAACTAATCCCGCCGATCCAAATTACTAAT</td></tr>
<tr><td>|NC_001664.2| Human herpesvirus 6A</td><td>/5Biosg/AAACTTTACTCATGGTTTCTGCACGTTCTCACTTTCGAAGACATTCAGTTTCCAG</td></tr>
<tr><td>|NC_001664.2| Human herpesvirus 6A</td><td>/5Biosg/AAGTGGTAAACTTTAAAATCGGAGTGGTGTGTTCGGCGTCGGTGAATTTT</td></tr>
<tr><td>|NC_001664.2| Human herpesvirus 6A</td><td>/5Biosg/CGATGACCGCTAAATTGATAATAAGTCCTGTGCTCCACTCGGATTCAAAGAAA</td></tr>
<tr><td>|NC_001664.2| Human herpesvirus 6A</td><td>/5Biosg/TTAGCAATTTTTAAGGTTCTAACCCGCTTGGGTAGGAAAGACCCTAACCAC</td></tr>
<tr><td>|NC_001501.1| Moloney murine leukemia virus</td><td>/5Biosg/GATAGACAGGGAGGAGAACGAAGGAGGTCCCAACTCGATCGCGAC</td></tr>
<tr><td>|NC_001501.1| Moloney murine leukemia virus</td><td>/5Biosg/GTAACCAATGGAGATCGGGAGACGGTATGGGCAACTTCTGGCAAC</td></tr>
<tr><td>|NC_014480.2| Torque teno virus 2</td><td>/5Biosg/AAAACACAGAATCACAGTCAGAATAGGAGCCCCTAAAACCTTTGAGGACAAG</td></tr>
<tr><td>|NC_014480.2| Torque teno virus 2</td><td>/5Biosg/GAGACAGGAGACACAGCCATAAACACCAACGCTAGACTGACACTAATATGC</td></tr>
<tr><td>|NC_001583.1| Human papillomavirus type 26</td><td>/5Biosg/CCTATGTATGTTATTAAATGTGCCAGAAACGCAATTACTAATTGAACCACCAAAATTGCG</td></tr>
<tr><td>|NC_001583.1| Human papillomavirus type 26</td><td>/5Biosg/TGGTGCAATGGGCGTTCGATCATGACATAACAGATGATAGTGAAATTGCATTTAAATATG</td></tr>
<tr><td>|NC_015961.1| Tomato chlorotic leaf distortion virus</td><td>/5Biosg/TATGGCTCTCTCTCTTCTGTCAACGTGGCGCATTATAGACCATGGTCTTAAATAA</td></tr>
<tr><td>NC_010729.1| *Porphyromonas gingivalis*</td><td>/5Biosg/GGGGAGAGGTAAGAGGACTTAAGGAGCAGGAACTTATTGAATTAGAAATAAGTATATGCA</td></tr>
<tr><td>|AY689230.1| *Prevotella nigrescens*</td><td>/5Biosg/CAACGGCCCACCAAGGCGACGATCAGTAGGGGTTCTGAGAGGAAG</td></tr>
<tr><td>|AY689230.1| *Prevotella nigrescens*</td><td>/5Biosg/TGTTGTGAAATTTAGGTGCTCAACATTTAACTTGCAGCGCGAACTGTCAGACTT</td></tr>
<tr><td>|AY689230.1| *Prevotella nigrescens*</td><td>/5Biosg/TAATGCAAGTTGCATCCAATCTTGAAATCCGGTCCCAGTTCGGACT</td></tr>
<tr><td rowspan="4">HPV16 pool</td><td>|NC_001526.2| Human papillomavirus type 16</td><td>/5Biosg/TGCAAAGGCAGCAATGTTAGCAAAATTTAAAGAGTTATACGGGGTGAGTTTTTCAGAATT</td></tr>
<tr><td>|NC_001526.2| Human papillomavirus type 16</td><td>/5Biosg/CAACGAAGTATCCTCTCCTGAAATTATTAGGCAGCACTTGGCCAACCAC</td></tr>
<tr><td>|NC_001526.2| Human papillomavirus type 16</td><td>/5Biosg/CCTTGGGCACCGAAGAAACACAGACGACTATCCAGCGACCAAGAT</td></tr>
<tr><td>|NC_001526.2| Human papillomavirus type 16</td><td>/5Biosg/GGAATTTTGGTCTACAACCTCCCCCAGGAGGCACACTAGAAGATA</td></tr>
</table>

Table S4

Accession	Library	Bcounts	Bcounts.probe	Bcounts.not.pro	Fcounts	Fcounts.probe	Fcounts.not.pro	HPVcounts	HPVcounts.probe	HPVcounts.not.probe	Ocounts	Ocounts.probe	Ocounts.not.pro	Pcounts	Pcounts.probe	Pcounts.not.pro	Chr	Start	End	IGV_coor
gi|437738|emb|X73952.1| A.haemolyticum gene for 16S rRNA	B Library	80	64	16	1	0	1	1	0	1	0	0	0	0	0	0	chr112	33236	34723	chr112:33236-34723
gi|642969|emb|X78719.1| Actinomyces radingae 16S rRNA gene, starin APL1	B Library	33	19	14	0	0	0	1	0	1	0	0	0	0	0	0	chr112	42514	43941	chr112:42514-43941
gi|642970|emb|X78720.1| Actinomyces turicensis 16S rRNA gene, strain APL10	B Library	232	166	66	3	2	1	1	0	1	0	0	0	0	0	0	chr112	44043	45494	chr112:44043-45494
gi|1838945|emb|X82451.1| A.meyeri 16S rRNA gene	B Library	267	207	60	7	5	2	1	0	1	0	0	0	1	1	0	chr112	65530	66969	chr112:65530-66969
gi|1838948|emb|X97049.1| Arcanobacterium phocae 16S rRNA gene	B Library	108	80	28	0	0	0	1	0	1	0	0	0	0	0	0	chr112	67071	68485	chr112:67071-68485
gi|12831925|emb|AJ250959.1| Arcanobacterium sp. partial 16S rRNA gene, strain M430/94/2	B Library	136	101	35	0	0	0	1	0	1	0	0	0	0	0	0	chr112	121510	122984	chr112:121510-122984
gi|16304874|emb|AJ416941.1| Mycobacterium chelonae partial 16S rRNA gene, strain MB834	B Library	901	859	42	2	0	2	0	0	0	0	0	0	1	0	1	chr113	4537160	4538597	chr113:4537160-4538597
gi|19387328|gb|AF480601.1| Mycobacterium kansasii 16S ribosomal RNA gene, partial sequence	B Library	520	510	10	0	0	0	0	0	0	0	0	0	0	0	0	chr114	3299820	3301287	chr114:3299820-3301287
gi|40645143|dbj|AB091200.1| Frateuria aurantia gene for 16S rRNA, partial sequence, strain:IFO13331	B Library	24	12	12	0	0	0	0	0	0	1	1	0	2	0	2	chr120	2491903	2493455	chr120:2491903-2493455
gi|42529116|gb|AY457068.1| Mycobacterium fortuitum subsp. fortuitum strain ATCC 49404 16S ribosomal RNA gene, part	B Library	2271	2242	29	1	0	1	0	0	0	2	2	0	0	0	0	chr120	2493557	2495038	chr120:2493557-2495038
gi|56542013|emb|AJ717364.1| Rothia dentocariosa 16S rRNA gene, isolate CV54	B Library	428	111	317	8	4	4	2	0	2	0	0	0	5	5	0	chr124	1197789	1199293	chr124:1197789-1199293
gi|59889718|emb|AJ879697.1| Arcanobacterium sp. 106-04 16S rRNA gene, isolate 106-04	B Library	73	37	36	0	0	0	1	0	1	0	0	0	1	0	1	chr124	1204136	1205579	chr124:1204136-1205579
gi|170679574|ref|NC_010498.1| Escherichia coli SMS-3-5, complete genome	B Library	156	156	0	24	24	0	4	4	0	8	8	0	37	37	0	chr146	3422056	8490443	chr146:3422056-8490443
gi|194326193|emb|FM180525.1| Brevundimonas diminuta partial 16S rRNA gene, strain B11	B Library	275	245	30	0	0	0	0	0	0	0	0	0	0	0	0	chr151	5312988	5314405	chr151:5312988-5314405
gi|454440|dbj|D14147.1|PEP16SRR12 Peptoniphilus indolicus gene for 16S ribosomal RNA, partial sequence	B Library	942	938	4	0	0	0	0	0	0	0	0	0	0	0	0	chr153	9960082	9961452	chr153:9960082-9961452
gi|34787154|emb|AJ576084.1| Mobiluncus curtisii 16S rRNA gene, strain CCUG 22339	B Library	23	12	11	0	0	0	0	0	0	0	0	0	0	0	0	chr153	9997469	9998968	chr153:9997469-9998968
gi|62484792|emb|AJ937773.1| Propionibacterium jensenii 16S rRNA gene, strain NCFB 571	B Library	49	45	4	0	0	0	0	0	0	0	0	0	0	0	0	chr154	6299	7788	chr154:6299-7788
gi|19908237|gb|AY083223.1| Malassezia furfur strain NCPF 3349 18S ribosomal RNA gene, partial sequence	F Library	271	1	270	4679	2423	2256	22	0	22	27	0	27	2868	0	2868	chr156	332527	334155	chr156:332527-334155
gi|134147422|gb|EF119339.1| Pleistophora mulleri 16S ribosomal RNA gene, partial sequence; internal transcribed spacer, co	F Library	20	16	4	2057	2002	55	0	0	0	8	6	2	142	1	141	chr156	552700	556396	chr156:552700-556396
gi|152716670|dbj|AB266392.1| Pneumocystis jirovecii gene for 18S rRNA, partial sequence	F Library	272	0	272	1432	35	1397	10	0	10	35	0	35	3765	13	3752	chr156	642413	644143	chr156:642413-644143
gi|160624242|gb|DS544748.1| Coccidioides posadasii CPA 0001 supercont1.256 mitochondrial scaffold, whole genome sho	F Library	0	0	0	0	0	0	0	0	0	0	0	0	0	0	0	chr156	707249	781713	chr156:707249-781713
gi|165881900|gb|EU192364.1| Malassezia globosa 18S ribosomal RNA gene, partial sequence; and 18S ribosomal RNA gene	F Library	242	1	241	4599	2386	2213	18	0	18	27	0	27	2617	0	2617	chr156	810548	812084	chr156:810548-812084
gi|165881902|gb|EU192366.1| Malassezia pachydermatis strain ATCC 14522 18S ribosomal RNA gene, partial sequence	F Library	245	1	244	4465	2228	2237	19	0	19	25	0	25	2639	1	2638	chr156	813849	815404	chr156:813849-815404
gi|165881903|gb|EU192367.1| Malassezia restricta strain CBS 7877 18S ribosomal RNA gene, partial sequence; and 18S ribo	F Library	246	1	245	4646	2423	2223	19	0	19	23	0	23	2637	0	2637	chr156	815506	817053	chr156:815506-817053
gi|165881904|gb|EU192368.1| Malassezia slooffiae strain CBS 7956 18S ribosomal RNA gene, partial sequence	F Library	264	1	263	4647	2392	2255	21	0	21	27	0	27	2760	0	2760	chr156	817155	818695	chr156:817155-818695
gi|165881905|gb|EU192369.1| Malassezia sympodialis 18S ribosomal RNA gene, partial sequence	F Library	251	1	250	4630	2377	2253	20	0	20	24	0	24	2638	1	2637	chr156	818797	820343	chr156:818797-820343
gi|3033522|gb|AF044392.1| Pleistophora ehrenbaumi small subunit ribosomal RNA gene, partial sequence; internal transcribe	F Library	1	0	1	1	1	0	0	0	0	0	0	0	3	0	3	chr156	1178952	1180347	chr156:1178952-1180347
gi|56377808|dbj|AB126649.1| Rhodotorula laryngis gene for 18S rRNA, partial sequence	F Library	17	0	17	74	61	13	0	0	0	3	0	3	23	2	21	chr157	475386	477166	chr157:475386-477166
gi|310698439|ref|NC_001526.2| Human papillomavirus type 16, complete genome	HPV16 Library	20	0	20	131	7	124	2311	1286	1025	1121	485	636	10	11	-1	chr108	2428642	2436545	chr108:2428642-2436545
gi|109390403|ref|NC_008186.1| Mouse parvovirus 2, complete genome	O Library	0	0	0	0	0	0	0	0	0	10	10	0	0	0	0	chr101	939921	944675	chr101:939921-944675
gi|12657941|ref|NC_002657.1| Classical swine fever virus, complete genome	O Library	0	0	0	0	0	0	0	0	0	5	5	0	0	0	0	chr103	121244	133543	chr103:121244-133543
gi|9628642|ref|NC_001699.1| JC polyomavirus, complete genome	O Library	0	0	0	0	0	0	0	0	0	75	75	0	0	0	0	chr106	4228998	4234126	chr106:4228998-4234126
gi|224020395|ref|NC_001664.2| Human herpesvirus 6A, complete genome	O Library	36	0	36	0	0	0	10	0	10	140	99	41	36	0	36	chr107	5835383	5994703	chr107:5835383-5994703
gi|9626958|ref|NC_001501.1| Moloney murine leukemia virus, complete genome	O Library	1	0	1	0	0	0	0	0	0	27	27	0	0	0	0	chr108	2207767	2216097	chr108:2207767-2216097
gi|312192953|ref|NC_014480.2| Torque teno virus 2, complete genome	O Library	0	0	0	0	0	0	0	0	0	1	0	1	0	0	0	chr108	4647569	4650889	chr108:4647569-4650889
gi|9627305|ref|NC_001583.1| Human papillomavirus type 26, complete genome	O Library	1	0	1	1	0	1	5	0	5	62	61	1	0	0	0	chr109	535128	542981	chr109:535128-542981
gi|61602086|ref|NC_006879.1| Simian adenovirus 1, complete genome	O Library	0	0	0	0	0	0	0	0	0	27	27	0	0	0	0	chr110	2596095	2630543	chr110:2596095-2630543
gi|350528582|ref|NC_015961.1| Tomato chlorotic leaf distortion virus-[Venezuela:Zulia:2004] DNA_B, complete genome	O Library	0	0	0	0	0	0	0	0	0	1	1	0	0	0	0	chr110	4793159	4795762	chr110:4793159-4795762
gi|188993864|ref|NC_010729.1| Porphyromonas gingivalis ATCC 33277, complete genome	O Library	737	0	737	4	0	4	4	0	4	10	2	8	14	0	14	chr150	2	2354886	chr150:2-2354886
gi|51339823|gb|AY689230.1| Prevotella nigrescens strain ChDC B270 16S ribosomal RNA gene, partial sequence	O Library	185	101	84	0	0	0	0	0	0	55	54	1	2	2	0	chr154	4703	6197	chr154:4703-6197
gi|2772639|gb|U94382.1|U94382 Toxocara canis 18S small subunit ribosomal RNA gene, partial sequence	P Library	368	0	368	1286	0	1286	12	0	12	24	0	24	1457	5	1452	chr156	1160245	1161997	chr156:1160245-1161997
gi|5565950|gb|AF151921.1|AF151921 Prosthodendrium longiforme 28S ribosomal RNA gene, partial sequence	P Library	89	0	89	230	0	230	3	0	3	41	0	41	9115	2	9113	chr156	1252386	1253592	chr156:1252386-1253592
gi|5565951|gb|AF151922.1|AF151922 Prosthodendrium hurkovaae 28S ribosomal RNA gene, partial sequence	P Library	118	65	53	289	165	124	6	5	1	56	27	29	12660	7263	5397	chr156	1253694	1254898	chr156:1253694-1254898
gi|6467291|gb|AF124475.1| Hymenolepis diminuta 18S small subunit ribosomal RNA gene, complete sequence	P Library	2006	0	2006	987	23	964	22	0	22	239	0	239	1470	123	1347	chr157	16149	18321	chr157:16149-18321
gi|32264723|gb|AY245759.1| Centrocestus formosanus 18S ribosomal RNA gene, partial sequence	P Library	3115	2	3113	1109	0	1109	24	0	24	232	0	232	1641	57	1584	chr157	264119	265949	chr157:264119-265949
gi|51704552|gb|AY702702.1| Contracaecum microcephalum 18S ribosomal RNA gene, partial sequence	P Library	398	2	396	1354	3	1351	12	0	12	23	0	23	1700	29	1671	chr157	462116	463844	chr157:462116-463844
gi|57545938|gb|AY851256.1| Trichinella nativa 18S ribosomal RNA gene, complete sequence	P Library	10954	1	10953	1662	4	1658	74	1	73	283	2	281	22880	14582	8298	chr157	486472	488295	chr157:486472-488295
gi|57545939|gb|AY851257.1| Trichinella britovi 18S ribosomal RNA gene, complete sequence	P Library	10815	1	10814	1624	4	1620	70	1	69	275	2	273	22802	14597	8205	chr157	488397	490220	chr157:488397-490220
gi|57545941|gb|AY851259.1| Trichinella murrelli 18S ribosomal RNA gene, complete sequence	P Library	11238	1	11237	1494	4	1490	70	1	69	278	2	276	20850	14585	6265	chr157	492251	494074	chr157:492251-494074
gi|57545942|gb|AY851260.1| Trichinella sp. T6 18S ribosomal RNA gene, complete sequence	P Library	12077	1	12076	1676	4	1672	74	1	73	290	2	288	23014	14584	8430	chr157	494176	495999	chr157:494176-495999
gi|57545944|gb|AY851262.1| Trichinella sp. T8 18S ribosomal RNA gene, complete sequence	P Library	11929	1	11928	1613	4	1609	72	1	71	282	2	280	22920	14599	8321	chr157	498027	499850	chr157:498027-499850
gi|57545945|gb|AY851263.1| Trichinella papuae 18S ribosomal RNA gene, complete sequence	P Library	13122	1	13121	1476	4	1472	74	1	73	296	2	294	20958	14587	6371	chr157	499952	501777	chr157:499952-501777
gi|57545946|gb|AY851264.1| Trichinella zimbabwensis 18S ribosomal RNA gene, complete sequence	P Library	13511	1	13510	1528	4	1524	73	1	72	292	2	290	22910	14599	8311	chr157	501879	503704	chr157:501879-503704
